# Structure-Activity Analysis Reveals Perturbed Cilia-Jun N-Terminal Kinase Signaling in MAPKBP1-Associated Kidney Disease

**DOI:** 10.1016/j.ekir.2025.05.049

**Published:** 2025-06-04

**Authors:** Christin Findeisen, Maria Papazian, Linda Pöschla, Anastasia Ertel, Wenjun Jin, Nydia Panitz, Elena Hantmann, Paul Coucke, Firdous Abdulwahab, Lama AlAbdi, Fawzan S. Alkuraya, May Salem, Hamad Alzaidan, Kai-Uwe Eckardt, Søren T. Christensen, Alexandre Benmerah, Sophie Saunier, Jan Halbritter, Ria Schönauer

**Affiliations:** 1 Division of Nephrology, University of Leipzig Medical Center, Leipzig, Germany; 2 Department of Nephrology and Medical Intensive Care, Charité - Universitätsmedizin Berlin, Berlin, Germany; 3 Department of Anesthesiology and Intensive Care Medicine, University Hospital Essen, University Duisburg-Essen, Essen, Germany; 4 Department of Biomolecular Medicine, University Hospital Ghent, Ghent, Belgium; 5 Department of Translational Genomics, Center for Genomic Medicine, King Faisal Specialist Hospital and Research Center, Riyadh, Saudi Arabia; 6 Department of Pediatrics, King Faisal Specialist Hospital and Research Center, Jeddah, Saudi Arabia; 7 Department of Medical Genomics, Centre for Genomic Medicine, King Faisal Specialist Hospital and Research Center, Riyadh, Saudi Arabia; 8 Department of Biology, University of Copenhagen, Copenhagen, Denmark; 9 Laboratory of Hereditary Kidney Diseases, Imagine Institute, Université Paris Cité, INSERM UMR 1163, Paris, France

**Keywords:** Cilia, MAPKBP1, nephronophthisis

## Abstract

**Introduction:**

Nephronophthisis (NPH) is a renal ciliopathy characterized by chronic tubulointerstitial fibrosis. Despite discovery of multiple disease genes, mechanisms of NPH-associated kidney degeneration remain poorly understood. In this study, we present details of clinical and molecular mechanisms of *MAPKBP1* (*NPHP20*) loss-of-function.

**Methods:**

This study was a systematic clinical and *in vitro* analysis of all published and newly identified cases using overexpression systems, patient fibroblasts, and mitogen-activated protein kinase binding protein 1 (MAPKBP1) knock down cells.

**Results:**

We demonstrated that *MAPKBP1*-NPH follows a distinct natural history, characterized by predominantly nonsyndromic kidney disease and exceptionally slow progression. Furthermore, we showed that endogenous MAPKBP1 is lost from ciliary basal bodies in patients with *NPHP20* and in *MAPKBP1* knock down, accompanied by shortened primary cilia. Overexpression of MAPKBP1 patient variants revealed impaired microtubule, centrosomal, and basal body localization. We propose that the activation status of Jun N-terminal kinase (JNK) determines the switch between centriolar association or dissociation of MAPKBP1 via distinct protein domains. Importantly, we found that JNK activation leads to the disassembly of cilia concomitantly with MAPKBP1 dissociation from the basal body. Downstream, we observed that impaired trafficking of phosphorylated JNK upon loss of MAPKBP1 and pharmacological disassembly of the JNK-target, actin, restores ciliary length in patients with *NPHP20*. Overall, MAPKBP1-associated molecular alterations appeared to be relatively modest, in line with late onset kidney function decline in patients with *NPHP20*.

**Conclusion:**

In summary, we propose alterations in cilia-related JNK pathways as a novel mechanism in the development of NPH. Thus, a more detailed investigation of JNK signaling and involved protein interactions are promising for the discovery of novel targets for urgently needed treatment strategies in NPH.

## Graphical abstract

**Figure:**
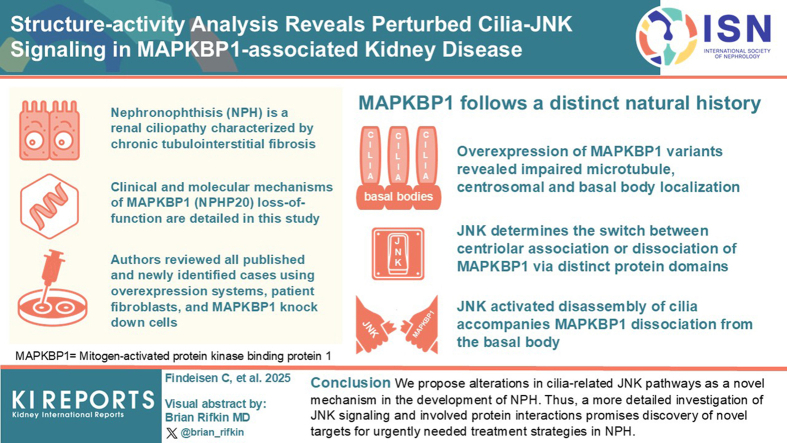

NPH; MIM #617271 is a rare autosomal recessive kidney disease characterized by progressive tubulointerstitial nephritis and renal fibrosis.^1^ With a prevalence of up to 1:50,000, NPH represents the most frequent cause of kidney failure (KF) in childhood and adolescence. More than 20 disease-related genes have been identified, most of which are involved in primary ciliary structure or function, which has led to the classification of NPH as a ciliopathy. The primary cilium is a microtubule-based membrane protrusion assembled from the mother centriole of the centrosomes in quiescent cells, which plays a crucial role as a cellular signaling hub via integration and transduction of a plethora of mechanical and chemical stimuli. The heterogeneity of subcellular localizations and associated signaling pathways reported for NPH-associated proteins (NPHPs) is reflected by pronounced phenotypic differences, regarding age of onset, disease progression, and extent of extrarenal manifestation.^2^ One of the newest members of the NPH family of proteins is MAPKBP1/NPHP20 (MAPKBP1/JNKBP1), a poorly characterized multidomain JNK-binding protein of almost unknown physiological function.^3^ In the context of NPH, MAPKBP1 was first described to be located only at the mitotic spindle poles but not at primary cilia and, thus, MAPKBP1 variation has been initially thought to represent a cilia-independent form of NPH.^4^ Recently, however, by the use of overexpression cell models, we found wild type (WT) MAPKBP1 to be localized to the interphase microtubule network and to both centrioles of centrosomes and ciliary basal bodies.^5^ Moreover, we showed that 2 C-terminally truncated NPH-related patient variants lose their homodimerization capabilities, which is a requirement for correct intracellular targeting.^5^ Based on the hypothesis that *MAPKBP1*-associated NPH requires a reclassification into the group of cilia-dependent NPH, we investigated ciliary consequences of MAPKBP1 patient variants. We elucidated structure-activity relationships that determine the crosstalk between MAPKBP1 and the primary cilium. In addition, we aimed to reevaluate all published cases, along with 1 newly identified case, to enable clinical cross-comparison with the broader NPH spectrum. A deeper understanding of both the natural history and the disease mechanisms of distinct genetic alterations will help to pinpoint shared mechanisms within defined NPH patient subgroups and may enable the identification of drug targets.

## Methods

### Patient Consent

Written informed consent from individuals NPHP20_I, II, V, and VII was previously obtained and approved by the Institutional Review Board at the Boston Children’s Hospital as reported.^4^^,^^5^ New Saudi Arabian patients were consented under KFHSRC IRB protocol (RAC 2210029).

### Transfection of Hela and H69 Cells for Immunofluorescence Microscopy

HeLa and H69 cells were seeded on 8-well μ-slides (ibidi, Martinsried, Germany), grown to 70% confluency and transfected with 250 to 500 ng DNA using Lipofectamin2000 (for HeLa cells, Invitrogen, Thermo Fisher Scientific, Waltham, MA) or FuGene (for H69 cells, Promega, Madison, WI) according to the manufacturer’s protocol (1 μl/μg and 3 μl/μg DNA for Lipofectamine2000 and FuGene, respectively). On the next day, fixation, permeabilization, and blocking were performed using the Image-iT Fixation/Permeabilization Kit (Thermo Fisher Scientific, Waltham, MA).

### Small Interfering RNA Knockdown

RPE1 cells were cultivated on 8-well μ-slide (ibidi, Martinsried, Germany), grown to 80% confluency and transfected with 100 nM small interfering (siRNA) targeting human *MAPKBP1* or scrambled siRNA (OriGene, Rockville, MD) using Lipofectamine 3000 according to the manufacturer’s protocol (Invitrogen, Thermo Fisher Scientific, Waltham, MA). Thirty-six hours after transfection, the medium was replaced by serum-reduced medium (DMEM/F-12 with 0.5% FBS) to induce cilia formation. After 24 hours, cells were fixed and stained as described above.

### Cell Treatments

For inhibition of JNK signaling, cells were treated with 10 μg/ml JNK Inhibitor VIII - CAS 894804-07-0 (Sigma Aldrich, St. Louis, MO) for 21 hours. Activation of JNK signaling was induced by incubation with 25 μg/ml anisomycin (AM) (#2222 Cell Signaling, Danvers, MA) or with 10 ng/ml human TGF-beta 1 (# PHG9204, Thermo Fisher Scientific, Waltham, MA) for 1 hour. Cells were treated with 0.1 μM Taxol (Invitrogen, Thermo Fisher Scientific, Waltham, MA) or 10 μg/ml nocodazole (Sigma Aldrich, St. Louis, MO) for 1 hour to stabilize or dissolve the microtubule cytoskeleton, respectively. Cells were treated with 250 nM cytochalasin D (Sigma Aldrich, St. Louis, MO) for 16 hours to dissolve actin filaments.

### Immunofluorescence Microscopy

Cells were incubated with primary and secondary antibodies (Supplementary Table S1) in phosphate-buffered saline (Thermo Fisher Scientific, Waltham, MA) at room temperature for 3 hours and 1 hour, respectively. Nuclei were stained with NucBlu Fixed Cell Ready Probes Reagent (Invitrogen, Thermo Fisher Scientific, Waltham, MA) and cells were covered with ibidi Mounting Medium. Cells were observed using an AxioObserver.Z1 with an ApoTome Imaging system (Carl Zeiss AG, Oberkochen, Germany. Fluorescence microscopy images were analyzed with the Zen light software (Carl Zeiss AG, Oberkochen, Germany). Intensities at the centrosome or basal body were quantified along a 10 μm line (5 μm = intensity at the centrosome or basal body, respectively, 0 μm = intensity in the cytosol). The intensity of JNK2 in the nucleus and cytosol was quantified as mean intensity inside a circle with 5 μm diameter.

### Statistical Analysis

Statistical analysis was performed using GraphPad Prism 9 (GraphPad Software, La Jolla, CA). Kaplan Meier analysis was used to determine median age for KF-free survival of patients with NPHP20. Quantification was based on mean values of at least 3 independent experiments. Statistical significance was calculated with *t* test and 1-way analysis of variance with Dunnetts correction (symbols used in Figure 1, Figure 2, Figure 3, Figure 4, Figure 5, Figure 6 and Supplementary Figures S1–S16: ∗*P* ≤ 0.05; ∗∗*P* ≤ 0.01; ∗∗∗*P* ≤ 0.001; and ∗∗∗∗*P* ≤ 0.0001).

### General Cell Culture Conditions, Preparation of Expression Vectors, Coimmunoprecipitation, and Western Blotting

Additional methods are described in the Supplementary Methods section. The antibody details are presented in the Supplementary Methods and Supplementary Table S2.

## Results

### The Genotypic and Phenotypic Landscape of MAPKBP1-associated NPH

**Figure 1:**
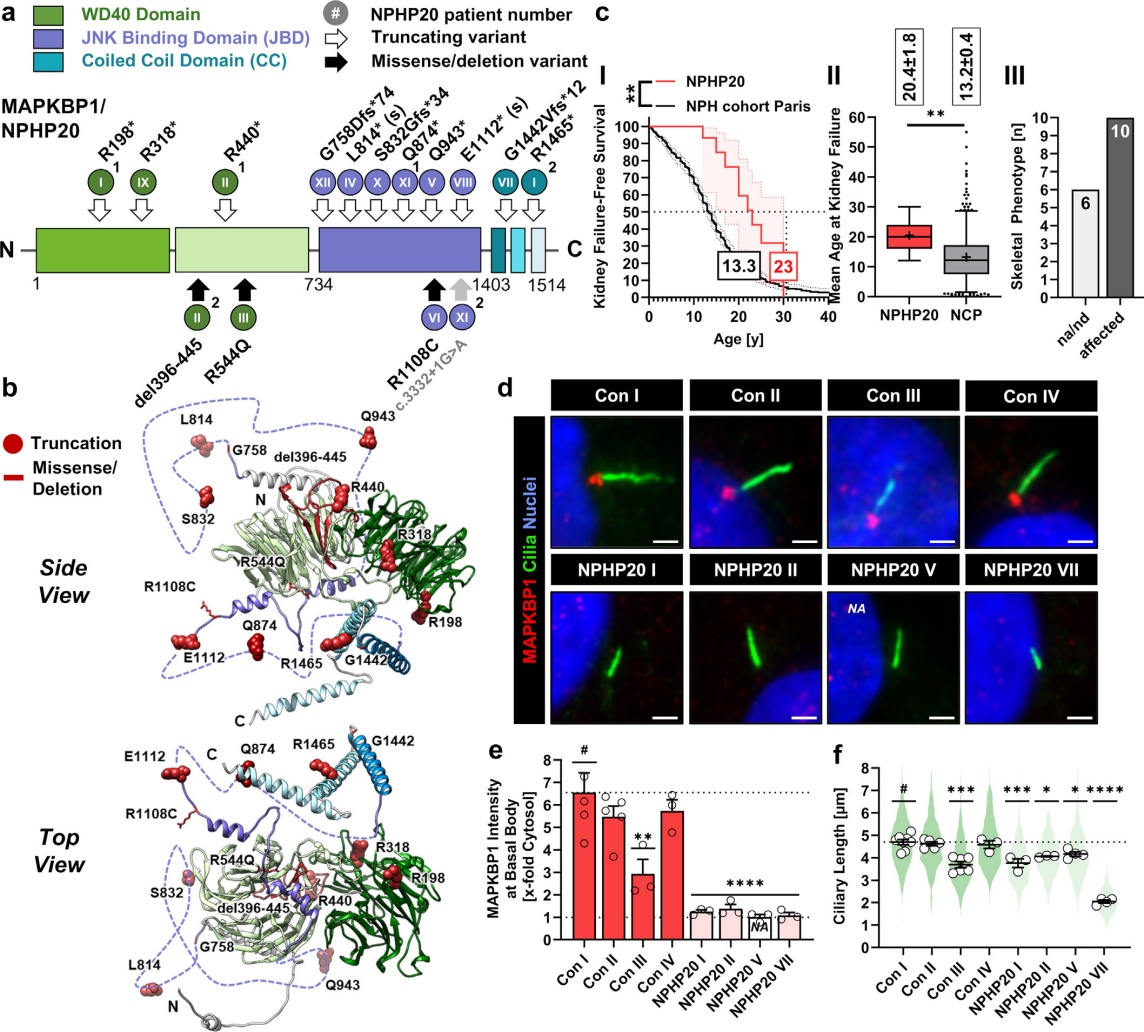
Genotypes and renal, extrarenal, and ciliary phenotypes of patients with NPHP20. (a) Domain scheme of MAPKBP1: protein domains are highlighted (WD40: green, JNK-binding domain [JBD]: violet, coiled-coil domain [CC]: blue), truncating and frameshift NPHP20 patient variants are represented with empty arrows, missense and deletion variants with black arrows. NPHP20 patient numbers (roman numbers in circles) correspond to Supplementary Table S3. Additional upper-case numbers indicate compound heterozygous patients (protein consequence of XI^2^ is unknown). (b) 3-dimensional structure of MAPKBP1 (alphafold AF-O60336-F1): positions of residues altered in patients with MAPKBP1 are highlighted in red (for truncating or frameshift variations the first altered residue is depicted as sphere; positions exchanged by missense or deletion alterations are highlighted as sticks). (c) Combined phenotypic characterization of published patients with NPHP20: (I) Kaplan-Meier analysis for kidney failure–free survival comparing patients with NPHP20 (*n* = 15) and aggregate data of patients with NPH from the Paris cohort (*n* = 502)^2^; (II) mean age at end-stage kidney disease (± SEM) for patients with NPHP20 (*n* = 9) versus patients from the Paris NPH cohort (*n* = 425)^2^; (III) number of patients with skeletal phenotype. (d) Immunofluorescence microscopy showing antibody staining of MAPKBP1 at the basal body of primary cilia in dermal fibroblast from healthy control individuals and patients with NPHP20 (MAPKBP1: red, acetylated α-tubulin: green, nuclei: blue). Scale bars equal 2 μm. (e) Quantification of MAPKBP1 intensities at the basal body in dermal fibroblast from healthy control individuals and patients with NPHP20 (mean ± SEM of at least 3 independent experiments). (f) Ciliary length in dermal fibroblast from healthy control individuals and patients with NPHP20. Black bordered white dots represent the mean of each independent experiment. Violin plots represent the sum of all individual datapoints. NA, not applicable (protein product devoid of antibody epitope); #, reference for statistical testing; ∗*P* ≤ 0.05; ∗∗*P* ≤ 0.01; ∗∗∗*P* ≤ 0.001; and ∗∗∗∗*P* ≤ 0.0001.

Since its discovery as NPH-associated gene in 2016,^4^ 16 patients from 12 families with biallelic loss-of-function variants in *MAPKBP1* have been reported (Figure 1a and b; Supplementary Table S3).^4^, ^5^, ^6^, ^7^, ^8^, ^9^ Together with another unpublished case in a cohort of 121 Saudi Arabian kidney disease patients (Supplementary Figure S1), we included 14 different genetic variations comprising 9 homozygous and 3 compound heterozygous combinations (Supplementary Table S3). At the protein level, the majority of variants lead to a C-terminal truncation of the MAPKBP1 protein (6 nonsense, 4 splice-site, 2 frameshift deletions). Only 2 homozygous missense variants were reported. MAPKBP1 is a 164 kDa protein with 3 separate domains (Figure 1a and b): the N-terminal part is predicted to fold into 2 separate 7-bladed WD40 propellers with unknown function (WD40 domain), followed by an unstructured region including the JNK-binding site (JNK-binding domain; JBD), and a C-terminal coiled-coil domain consisting of 3 alpha-helices, mediating homo- and heterodimerization (CC-domain).^4^^,^^5^^,^^10^ Interestingly, one of the missense variants as well as the in-frame deletion both affect the second WD40 propeller. Another missense variation is located within the JBD. We and others have shown that loss of the CC-domain leads to a disruption of dimerization and incorrect intracellular localization.^4^^,^^5^ Thus, truncating variants are likely disease-causing independent of their position within the protein.

Combined clinical end point analysis in all patients with *MAPKBP1*-associated kidney disease revealed a median age of 23 years and a mean age of 20.4 ± 1.8 years at KF (Figure 1cI and II, Supplementary Figure S2, Supplementary Table S3). Based on comparative data from the largest European NPH registry,^2^ patients with MAPKBP1 defects showed a significantly attenuated course with slower kidney function decline than all other known forms of NPH, suggesting progressive degeneration, rather than early-onset impairment (Supplementary Figure S2). Information on extrarenal manifestations of MAPKBP1-associated NPH is scarce. In contrast to other NPH-related ciliopathies, kidney disease is the predominant feature, often presenting as isolated, nonsyndromic chronic kidney disease with nonspecific atrophic morphology, occasional cysts, and severe tubulointerstitial fibrosis on kidney biopsy. However, 10 out of 16 patients presented with mild skeletal abnormalities, including loose patella (*n* = 1), short stature (*n* = 2), scoliosis (*n* = 5), or slight facial dysmorphisms (*n* = 2) (Figure 1cIII, Supplementary Table S3, Supplementary Figure S1). In contrast to *NPHP1*-associated kidney disease, which may similarly account for adult-onset KF (PMID: 29654215), retinal impairment was not part of the phenotypic spectrum in any of the published cases with MAPKBP1 deficiency (Supplementary Table S3).

### Endogenous MAPKBP1 Localizes to the Basal Body and is Related to Ciliary Length in Primary Fibroblasts

Initially, MAPKBP1 was observed at the mitotic spindle pole only and could neither be detected at the primary cilium in patient-derived fibroblasts or RPE1 cells.^4^ Recently, however, we observed green fluorescent protein (GFP)-tagged WT MAPKBP1 to localize to the ciliary basal body.^5^ This finding prompted us to reinvestigate 4 patient-derived fibroblasts NPHP20_I, NPHP20_II, NPHP20_V, NPHP20_VII (Supplementary Table S3)^5^ in comparison with fibroblasts obtained from 4 healthy controls (con I–IV). Indeed, staining with the original MAPKBP1-specific antibody showed basal body localization of MAPKBP1 in all healthy controls (Figure 1d and e; Supplementary Figure S3). Moreover, endogenous MAPKBP1 presented in the same specific 3 + 1 dotted pattern observed with GFP-overexpression plasmids,^5^ underlining the specificity of our observation. Strikingly, MAPKBP1 staining was completely absent in all of the patient-derived cell lines. No difference was observed for the percentage of ciliated cells; however, ciliary length measurements revealed overall minor but statistically significant shortening in individuals NPHP20 compared with controls (Figure 1f, Supplementary Table S4). Notably, 1 healthy control cell line (con III) showed a 50% decrease of MAPKBP1 at the ciliary base, which was associated with a significant reduction of ciliary length (Figure 1e and f; Supplementary Table S4). This observation led us to hypothesize that the amount of ciliary MAPKBP1 might be tightly regulated and directly related to the length of the primary cilium.

### Overexpression of NPH-Associated MAPKBP1 Variants Leads to Aberrant Intracellular Localization, Reduction of Primary Cilia, and Ciliary Shortening

**Figure 2:**
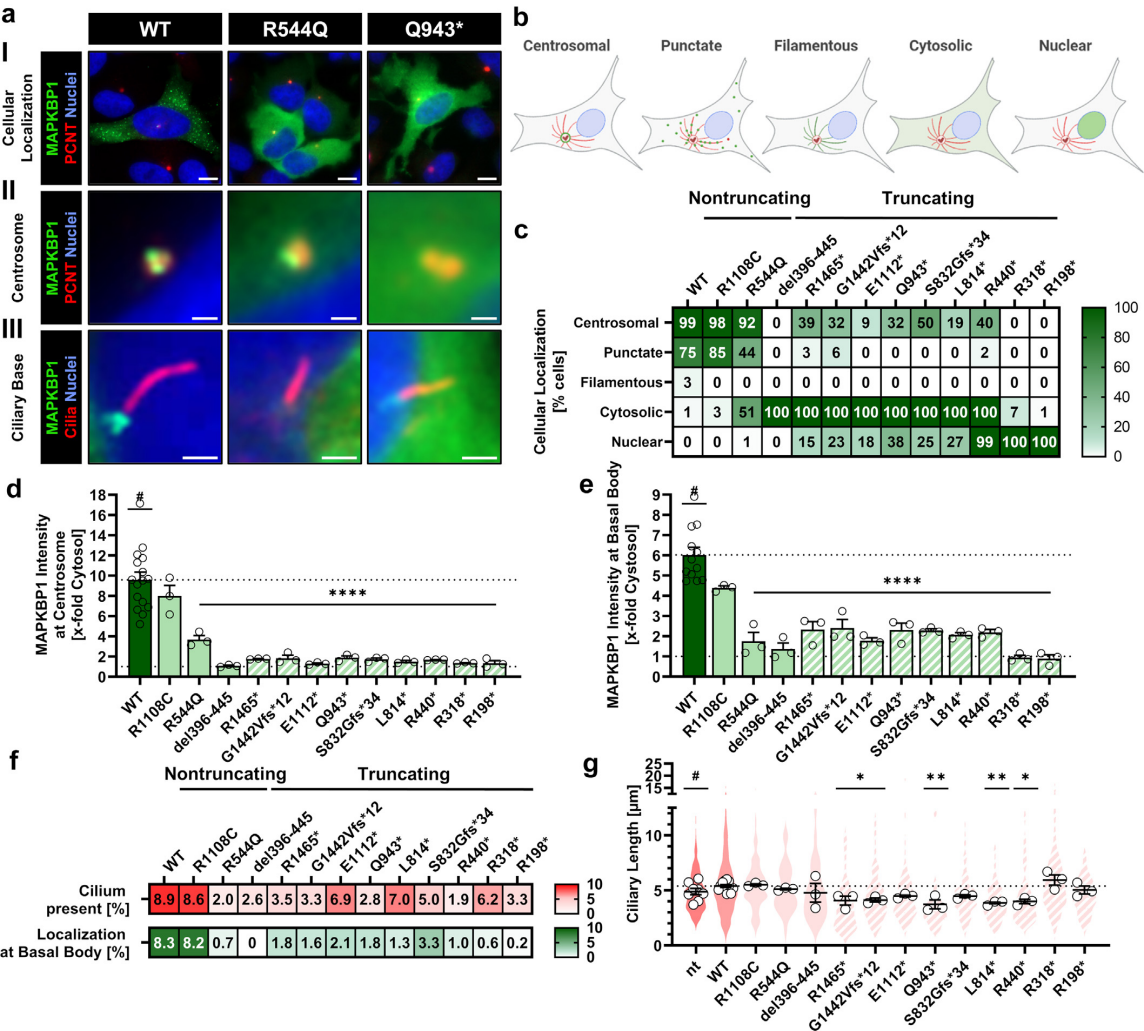
Intracellular localization and characterization of GFP-labeled WT MAPKBP1 and patient variants. (a) Immunofluorescence microscopy of HeLa (I and II) and H69 (III) cells transiently transfected with GFP-MAPKBP1 wild type or patient variant R544Q/Q943∗ as examples for missense or truncating mutations. Detailed microscopy images of all patient variants reported so far can be found in Supplementary Figures S3 to S5. Centrosomes and primary cilia were stained with anti PCNT or acetylated α-tubulin antibodies, respectively (GFP-MAPKBP1: green, PCNT/ acetylated α-tubulin: red, nuclei: blue). Representative example images are shown for the overall subcellular (I), centrosomal (II) and ciliary basal body (III) localization. Scale bars equal 10 μm (I); 1 μm (II); 2 μm (III). (b) Cartoon illustrating intracellular localization patterns of GFP-tagged MAPKBP1 variants (microtubule structures: red, MAPKBP1: green, nuclei: blue). (c) Quantification of cellular localization patterns for GFP-labeled MAPKBP1 WT and patient variants overexpressed in HeLa cells. (d) Quantification of GFP intensity at the centrosome relative to the cytosol of HeLa cells transiently transfected with GFP-MAPKBP1 variants. (e) Quantification of GFP intensity at the basal body of primary cilia relative to the cytosol of H69 cells transiently transfected with GFP-MAPKBP1 variants. (f) Quantification of transfected H69 cells presenting a primary cilium (red) and cells with GFP-MAPKBP1 localized at the basal body (green). Color scale ranges from 0% to 10% of transfected cells. Mean of at least 3 independent experiments. (g) Ciliary length of H69 cells transiently transfected with GFP-MAPKBP1 variants compared with nontransfected H69 cells. Black bordered white dots represent the mean of each independent experiment. Violin plots represent the sum of all individual datapoints. All data represent the mean (± SEM) of at least 3 independent experiments. Filled bars and violin plots: nontruncating patient variants, striped bars and violin plots: truncating patient variants. GFP, green fluorescent protein; PCNT, pericentrin; WT, wild type; #, reference for statistical testing; ∗*P* ≤ 0.05; ∗∗*P* ≤ 0.01; ∗∗∗*P* ≤ 0.001; and ∗∗∗∗*P* ≤ 0.0001.

To obtain a comprehensive picture of NPH-associated MAPKBP1 variation on the cellular level, we constructed GFP-fusions for all patient variants reported so far (NPHP20_XI^7^ and NPHP20_XII^6^ were published after study initiation). In nonciliated Hela cells, WT MAPKBP1 was observed predominantly at the centrosome and along interphase microtubules in a punctate or filamentous pattern, as previously described (Figure 2aI–II, b, and c; Supplementary Figures S4 and S5, Supplementary Table S5).^5^ In ciliated H69 cells, WT MAPKBP1 appeared in its characteristic 3 + 1 dotted centriolar pattern at the ciliary base (Figure 2aIII; Supplementary Figure S6). All truncating patient variants showed at least a 50% decrease of centrosomal localization, a major abrogation of microtubule association (punctate or filamentous) and an increase in cytosolic distribution (Figure 2aI–II, b, and c; Supplementary Figure S4 and S5, Supplementary Table S5). Loss of the CC-domain additionally led to a nuclear translocation of MAPKBP1, which was gradually increased with shortening of the protein. In contrast, nontruncating variants were not observed in the nucleus. Although R1108C localization was unaltered, R544Q showed a decrease in microtubule association and an increase in cytosolic abundance despite preserved centrosomal localization. Cells expressing del396-445 only showed cytosolic distribution, pointing toward pathogenicity for this variant despite presence of the CC-domain. Intensity measurements at the centrosome and ciliary basal body revealed a strong reduction of all truncated patient variants compared with the WT (Figure 2d and e; Supplementary Figures S5 and S6, Supplementary Table S6). Although not significant, R1108C showed a decrease at both structures, and R544Q as well as del396-445 were in a comparable range with truncated variants. In line with those findings, the number of transfected cells showed a decreased ciliation rate and ciliated cells were less likely to harbor MAPKBP1 at the basal body (Figure 2f; Supplementary Table S6). Finally, ciliary length measurements revealed a nonsignificant increase for WT-transfected cells compared with nontransfected conditions (Figure 2g; Supplementary Table S6). Overall, we observed a subtle reduction of ciliary length for the majority of truncated patient variants with 5 of 9 showing significant differences. All CC-containing constructs as well as the 2 shortest variants R318∗ and R198∗, however, had no impact on ciliary length, suggesting that ciliary number and length are differently affected by pathogenic MAPKBP1 variation.

### WD40 Propellers and JBD are Required for Basal Body Recruitment of MAPKBP1 in Addition to the CC-Domain

**Figure 3:**
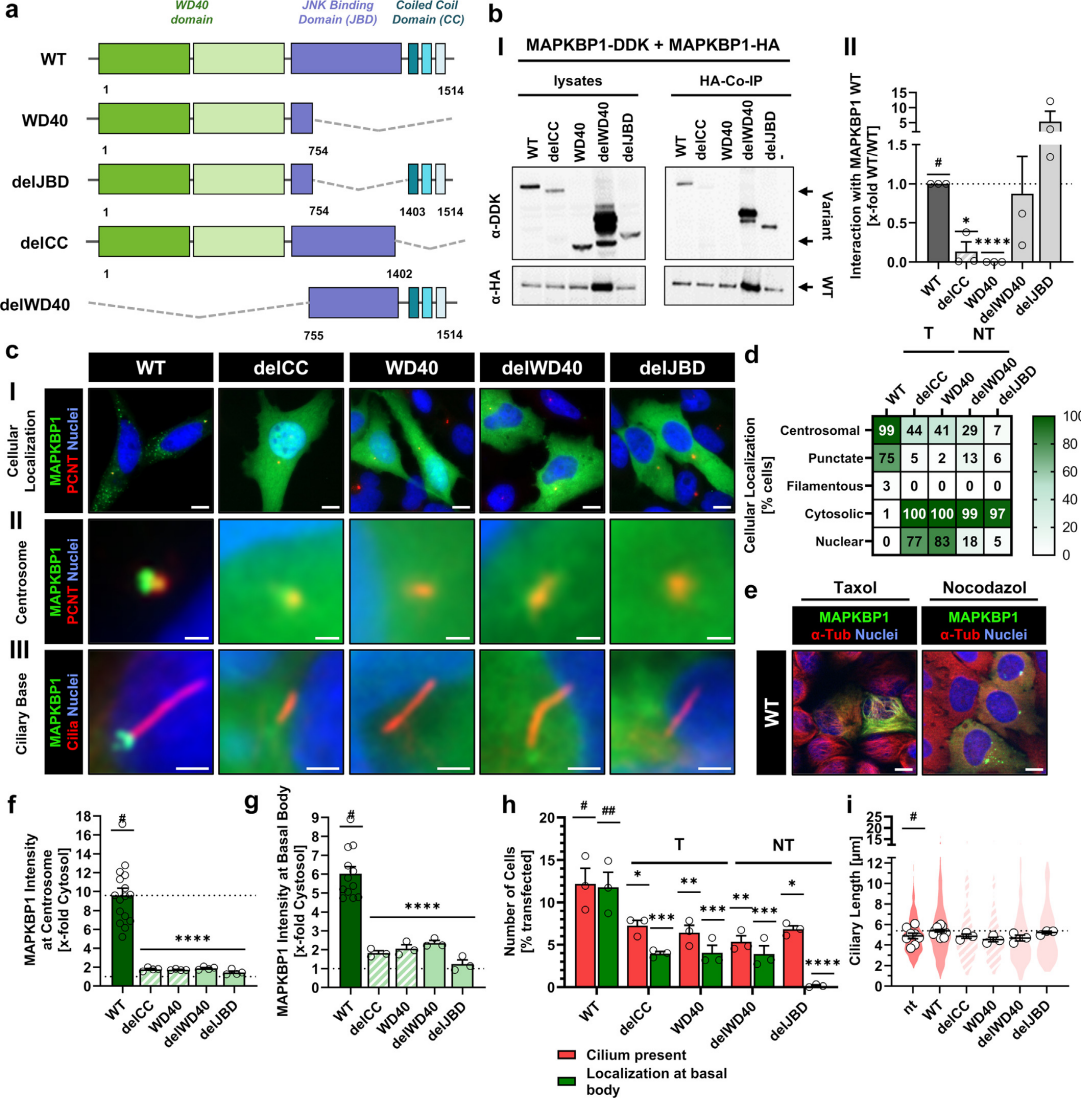
Impact of MAPKBP1 protein domains on intracellular trafficking and ciliary phenotype. (a) Schematic representation of the protein domains of WT MAPKBP1 and artificial deletion variants. Deleted regions are shown as dotted grey lines (WD40 domain: green, JNK-binding domain: violet, coiled-coil domain: blue). (b) Co-IP of DDK-tagged WT MAPKBP1 and HA-tagged deletion variants overexpressed in HEK293T cells. (I) Cell lysates and Co-IP eluates were analyzed with western blots using anti-HA and anti-DDK antibodies. (II) Analysis of homodimerization efficiency by quantification of western blot bands. (c) Immunofluorescence microscopy images showing HeLa (I and II) and H69 (III) cells transiently transfected with GFP-MAPKBP1WT or deletion variants. Centrosomes and primary cilia were stained with anti- PCNT or acetylated α-tubulin antibodies, respectively (GFP-MAPKBP1: green, PCNT/ acetylated α-tubulin: red, nuclei: blue). Representative example images are shown for the overall subcellular (I), centrosomal (II) and ciliary basal body (III) localization. Scale bars equal 10 μm (I); 1 μm (II); 2 μm (III). (d) Quantification of cellular localization patterns from GFP-labeled MAPKBP1 wild type and patient variants overexpressed in HeLa cells. (e) Immunofluorescence microscopy of HeLa cells transiently transfected with GFP-MAPKBP1 wild type and treated with Taxol or nocodazole, respectively. Microtubules were stained with anti-α tubulin primary antibody (GFP-MAPKBP1: green, α tubulin: red, nuclei: blue). Scale bars equal 10 μm. (f) Quantification of GFP intensity at the centrosome in HeLa cells transiently transfected with GFP-MAPKBP1 variants. (g) Quantification of GFP intensity at the basal body of primary cilia in H69 cells transiently transfected with GFP-MAPKBP1 variants. (h) Quantification of transfected H69 cells presenting a primary cilium (red) and cells with GFP-MAPKBP1 localized at the basal body (green). (i) Ciliary length of H69 cells transiently transfected with GFP-MAPKBP1 variants compared with nontransfected H69 cells. Black bordered white dots represent the mean of each independent experiment. Violin plots represent the sum of all individual data points. All data represent the mean (± SEM) of at least 3 independent experiments. Co-IP, co-immunoprecipitation, GFP, green fluorescent protein; HA, hemagglutinin; NT, nontruncating; PCNT, pericentrin; T, truncating; #, reference for statistical testing; ∗*P* ≤ 0.05; ∗∗*P* ≤ 0.01; ∗∗∗*P* ≤ 0.001; and ∗∗∗∗*P* ≤ 0.0001. Filled bars and violin blots denote nontruncating variants; striped bars and violin blots denote truncating variants.

We next investigated the regulatory requirements for correct intracellular distribution of MAPKBP1 with constructs either lacking or consisting of only one of the 3 MAPKBP1 protein domains (Figure 3a): delCC and WD40 as surrogates for truncating patient variants including and excluding the JBD, respectively; delJBD and delWD40 to dissect the function of both domains in presence of the CC-domain. Coimmunoprecipitation experiments with WT MAPKBP1 clarified that the CC-domain is not only necessary but also sufficient for homodimerization (Figure 3bI and II; Supplementary Table S7). Interestingly, all domain variants predominantly appeared cytosolic and showed a reduction in centrosomal, microtubular and ciliary localization (Figure 3cI–III and d; Supplementary Figures S4–S6, Supplementary Table S5). Treatment of cells with the microtubule stabilizer Taxol led to an increased microtubular association of the wildtype, whereas the microtubule destabilizer nocodazole led to a loss of filamentous structures and a disordered appearance of remaining puncta (Figure 3e; Supplementary Figure S7). None of the domain deletion constructs showed enhanced targeting to microtubules upon Taxol treatment, confirming the requirement of all MAPKBP1 domains for microtubule binding (Supplementary Figure S7). MAPKBP1 intensity at the centrosome and ciliary basal body was drastically reduced for all variants (Supplementary Figure 3f and g; Supplementary Table S6). Furthermore, transfection of deletion constructs led to a reduction in ciliation as well as MAPKBP1 at the basal body in ciliated cells (Figure 3h; Supplementary Table S6). Interestingly, delJBD showed the strongest loss of function in terms of centriolar targeting. As observed for patient variants, domain deletions lacking the CC-domain led to a reduction in ciliary length (Figure 3i; Supplementary Table S6). Thus, we hypothesized that all protein domains are required for correct intracellular targeting of MAPKBP1 and that JBD plays the most important role in centriolar association.

### The D-Domain Sequence in MAPKBP1 JBD Determines Interaction With JNK2

**Figure 4:**
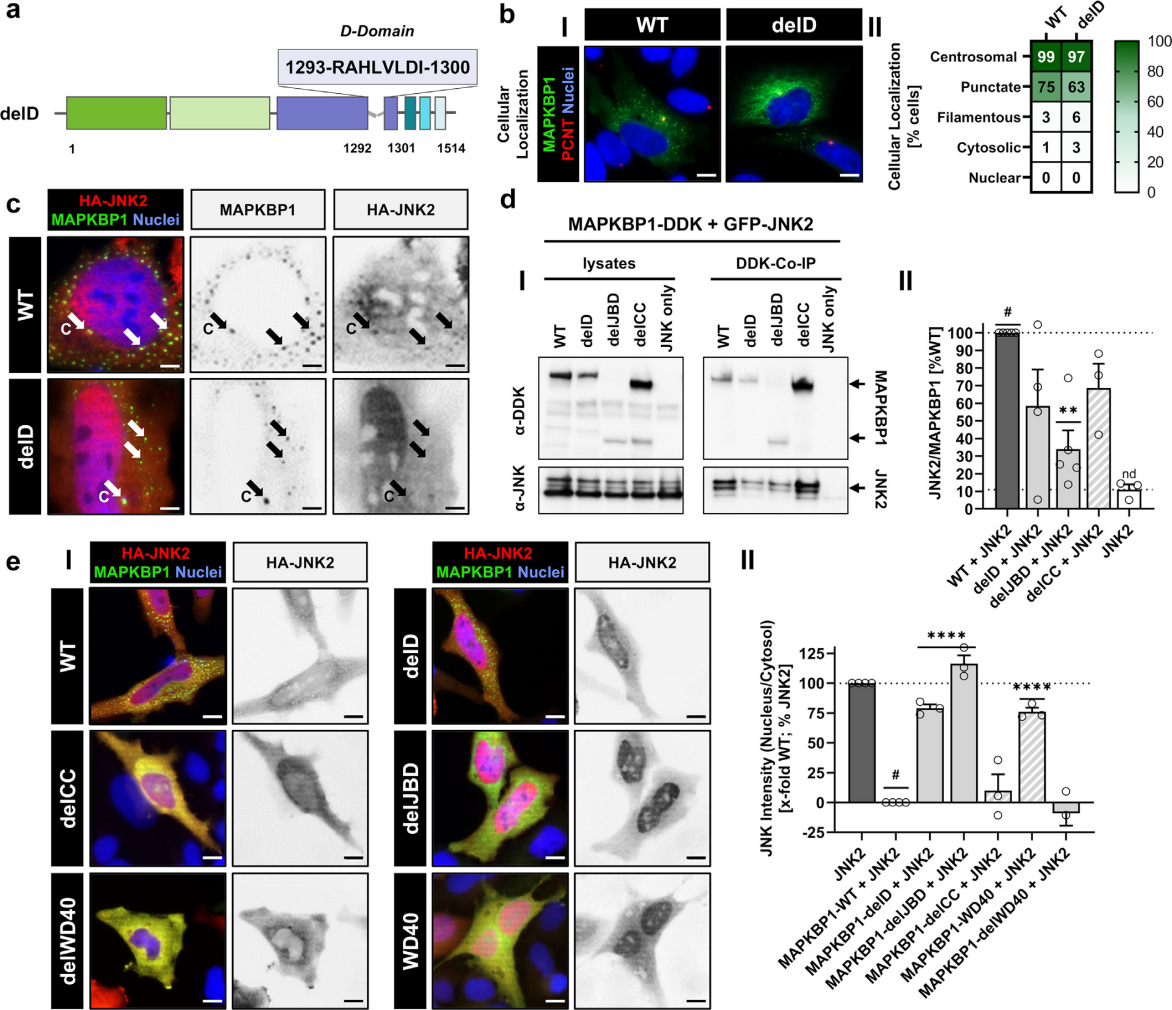
Importance of the D-domain for MAPKBP1-JNK interaction. (a) Schematic representation of MAPKBP1 lacking the D-domain. Amino acids 1293 to 1300 were deleted to generate MAPKBP1-delD variant. WD40 domain: green, JBD: purple, coiled-coil domain: blue. (b) Immunofluorescence microscopy of GFP- wild type MAPKBP1 compared with delD variant. (I) Centrosomes were stained with primary anti-PCNT antibody (MAPKBP1: green, PCNT: red, nuclei: blue). Scale bars equal 10 μm. (II) Quantification of cellular localization patterns comparing GFP-labeled MAPKBP1 wild type and delD-variant overexpressed in HeLa cells. (c) Immunofluorescence microscopy of HeLa cells transiently cotransfected with GFP-MAPKBP1 wild-type or delD, respectively, cotransfected with HA-JNK2 (MAPKBP1: green, JNK: red, nuclei: blue). Scale bars equal 5 μm. (d) Co-IP of DDK-tagged MAPKBP1 variants with GFP-tagged JNK2 cotransfected in HEK293T cells. (I) Cell lysates and Co-IP eluates detected with anti-DDK and anti-JNK antibodies. (II) Quantification of the western blot bands. (e) Immunofluorescence microscopy of HeLa cells transiently cotransfected with GFP-tagged MAPKBP1 variants and HA-tagged JNK2. (I) Representative images showing nuclear translocation of JNK2 (MAPKBP1: green, JNK2: red, nuclei: blue). Scale bars equal 10 μm. (II) Quantification of JNK2-intensity in the nucleus in relation to the cytosol depending on the co-transfected MAPKBP1 variant. All data represent the mean (± SEM) of at least 3 independent experiments. Co-IP, coimmunoprecipitation; GFP, green fluorescent protein; HA, hemagglutinin; PCNT, pericentrin;#, reference for statistical testing; ∗*P* ≤ 0.05; ∗∗*P* ≤ 0.01; ∗∗∗*P* ≤ 0.001; and ∗∗∗∗*P* ≤ 0.0001.

Because MAPKBP1's JBD is predicted to include the major JNK-binding site,^3^ we suspected JNK-interaction to play a role in intracellular regulation of MAPKBP1 similar to its paralog WDR62.^11^^,^^12^ Therefore, we generated a construct lacking the D-Domain, which is a JNK-binding consensus sequence located within the JBD (delD; Figure 4a).^13^ Interestingly, despite an increase in filamentous structures, which suggested an increased association to microtubules, intracellular distribution of MAPKBP1-delD was overall comparable to the wildtype (Figure 4bI-II; Supplementary Figures S4–S6, and S8; Supplementary Table S5 and S6). However, cotransfection of JNK2 revealed its colocalization with MAPKBP1 puncta only for WT but not for delD (Figure 4c). Furthermore, coimmunoprecipitation experiments showed a decrease in JNK interaction for delD and delJBD (Figure 4dI and II; Supplementary Table S8). MAPKBP1-JNK interaction was also slightly decreased for delCC, suggesting a small reduction in binding efficiency upon loss of homodimerization in line with previous findings in patient cells.^4^ Moreover, WT MAPKBP1 was able to exclude overexpressed JNK2 from the nucleus in contrast to variants lacking the D-Domain (Figure 4eI and II; Supplementary Table S9). These findings confirm JBD (and more specifically the D-Domain) to mediate the mutual interaction of MAPKBP1 and JNK2.

### Distinct Protein Domains Regulate Microtubular and Centriolar Association of MAPKBP1 Depending on JNK Activation

**Figure 5:**
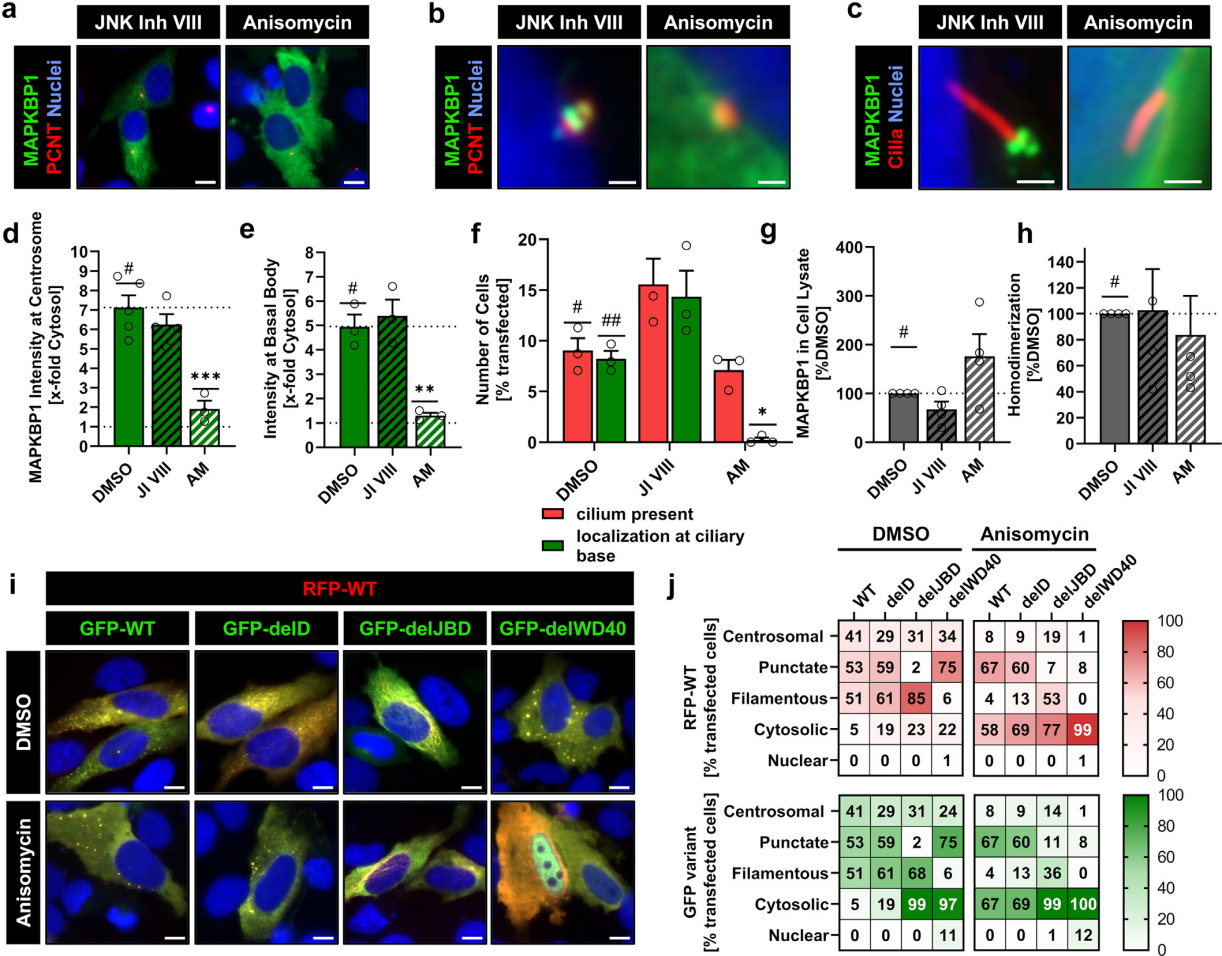
Influence of JNK signaling on MAPKBP1 localization to microtubular structures. (a–c) Immunofluorescence microscopy showing overall (a) intracellular and (b) centrosomal localization of GFP-tagged MAPKBP1 wild type in transiently transfected HeLa cells (GFP-MAPKBP1: green, PCNT: red, nuclei: blue) and localization of GFP-MAPKBP1 wild type at (c) the basal body of transiently transfected H69 cells (GFP-MAPKBP1: green, acetylated-α tubulin: red, nuclei: blue) upon either inhibition (JNK Inh VIII) or activation (anisomycin) of JNK signaling pathway. Scale bars equal (a) 10 μm; (b) 1 μm; (c) 2 μm. (d) Quantification of GFP intensity at the centrosome in HeLa cells transiently transfected with GFP-MAPKBP1 wild type treated with either JNK Inh VIII or anisomycin (AM), respectively. (e) Quantification of GFP intensity at the basal body relative to the cytosol of H69 cells transiently transfected with GFP-MAPKBP1 wild type treated with either JNK Inh VIII or anisomycin (AM), respectively. (f) Quantification of transfected H69 cells presenting a primary cilium (red) and cells with GFP-MAPKBP1 localized at the basal body (green) depending on treatment with either JNK Inh VIII or anisomycin (AM). (g) Quantification of MAPKBP1 wild type in cell lysates of HEK293T cells treated with either DMSO, JNK Inh VIII or anisomycin (AM), respectively. (h) Co immunoprecipitation to quantify homodimerization of MAPKBP1 wild type depending on JNK signaling activation (AM) or inhibition (JNK Inh VIII). (i) Immunofluorescence microscopy of HeLa cells transiently cotransfected with RFP-tagged MAPKBP1 wild type and GFP-tagged MAPKBP1 deletion variants. Images show influence of JNK activation (anisomycin) on homodimerization and cellular localization (RFP-MAPKBP1 wild type: red; GFP-MAPKBP1 variant: green, nuclei: blue). Scale bars equal 10 μm. (j) Quantification of cellular localization pattern depending on JNK signaling activation (anisomycin). All data represent the mean (± SEM) of at least 3 independent experiments. AM, Anisomycin; GFP, green fluorescent protein; JNK Inh VIII/JI VIII, JNK inhibitor VIII; PCNT, pericentrin; RFP, red fluorescent protein; #, ##, reference for statistical testing; ∗*P* ≤ 0.05; ∗∗*P* ≤ 0.01; ∗∗∗*P* ≤ 0.001; and ∗∗∗∗*P* ≤ 0.0001.

Next, we aimed to investigate the influence of JNK-signaling on MAPKBP1 subcellular localizations. Treatment of WT-transfected cells with JNK inhibitor VIII (JI VIII) for 21 hours had no effect on intensities of MAPKBP1 at centrosomes or basal bodies (Figure 5a–e; Supplementary Figure S9A–C; Supplementary Table S10). However, the proportion of ciliated cells and of cells with MAPKBP1 at basal bodies was slightly increased (Figure 5f, Supplementary Table S10). In contrast, 1 hour treatment with the JNK activator, AM revealed a complete loss of centrosomal and ciliary localization of MAPKBP1 and led to its cytosolic dispersal (Figure 5a–f; Supplementary Figure S9A–C). Furthermore, cell lysates showed a decreased and increased abundance of MAPKBP1 after treatment with JI VIII and AM, respectively, suggestive of a regulation of microtubular association depending on JNK activation (Figure 5g; Supplementary Table S11). Because cytosolic distribution was observed upon loss of the CC-domain,^5^ we tested homodimerization after JNK inhibition and activation. Coimmunoprecipitation experiments revealed a preserved dimerization upon AM treatment, excluding this mechanism as explanation for the observed phenomena (Figure 5h; Supplementary Table S11). In order to elucidate the structural requirements for JNK-dependent microtubular association, we cotransfected red fluorescent protein-labeled WT MAPKBP1 with the different GFP-labeled domain variants (Figure 5i and j; Supplementary Figure S10; Supplementary Table S12). Under control conditions, dimers consisting of delD and WT showed a decrease of centrosomal and punctate distribution, but a strong increase in filamentous patterns. This phenotype was even more pronounced in cells expressing WT/delJBD. In contrast, the WT/delWD40 combination led to a marked decrease in filamentous structures and a strong increase of intracellular puncta, which were larger than WT puncta and seemed to be dispersed within the cell without following microtubular structures similar to nocodazole treatment. Thus, we speculated that loss of 1 JBD, and a concomitant inhibition of JNK-binding, leads to a reduced microtubular dissociation, whereas loss of 1 WD40 domain is sufficient to inhibit efficient microtubular targeting. Indeed, treatment of cells with AM led to an increase of cytosolic distribution for all combinations, however, WT/delJBD retained a pronounced percentage of cells with filamentous structures compared with WT/WT (Figure 5i and j; Supplementary Figure S10; Supplementary Table S12). Moreover, WT homodimers presented increased amounts of large dyslocalized puncta, which was comparable to WT/delWD40 under control conditions. Taken together, those experiments suggest that MAPKBP1 homodimerization is required to provide a pair of WD40 domains that allows microtubular association and a pair of JBD for microtubular dissociation upon JNK activation.

### Endogenous MAPKBP1-JNK Interaction Impacts on Ciliary Length and pJNK Localization

**Figure 6:**
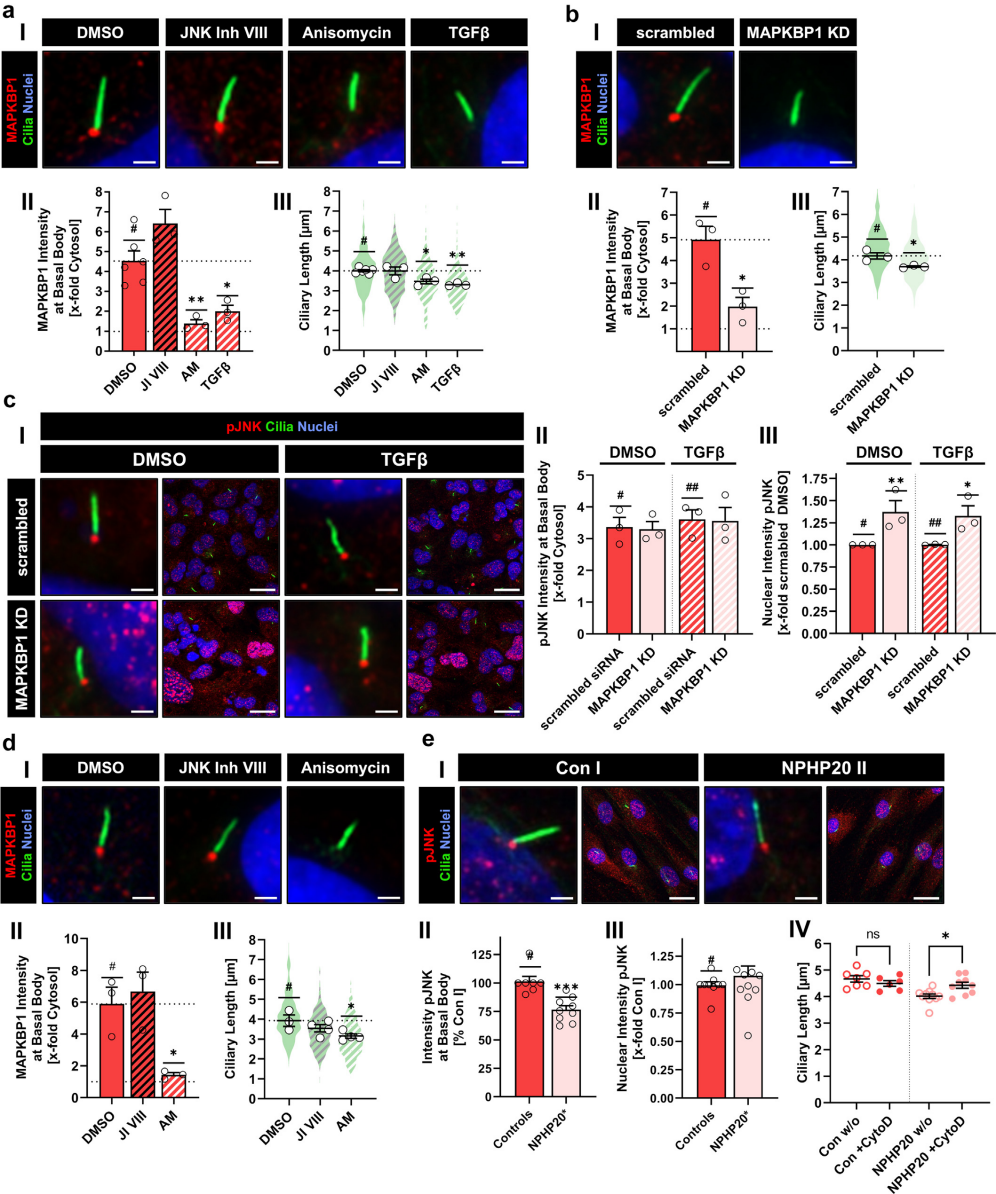
Mutual influence of JNK and MAPKBP1 in RPE1 cells and patient fibroblasts. (a) Immunofluorescence microscopy of RPE1 cells treated with either DMSO, JNK Inh VIII, anisomycin or TGFB. (b) Immunofluorescence microscopy of RPE1 cells transfected with anti-*MAPKBP1* siRNA (MAPKBP1-KD) or scrambled siRNA. (a–b I) Endogenous MAPKBP1 was stained with anti-MAPKBP1 antibody (MAPKBP1: red acetylated α-tubulin: green, nuclei: blue). Scale bars equal 2 μm. (a–b II) Quantification of MAPKBP1 intensity at the basal body. (a–b II) Ciliary length. Black bordered white dots represent the mean of each independent experiment. Violin plots represent the sum of all individual datapoints. (c) Immunofluorescence microscopy of RPE1 cells transfected with anti-*MAPKBP1* siRNA (MAPKBP1-KD) or scrambled siRNA and treated with either DMSO or TGFB. (I) Endogenous pJNK stained with anti-pJNK antibody (pJNK: red, acetylated α-tubulin: green, nuclei: blue). Scale bars equal 2 μm. (II) Quantification of pJNK intensity at the basal body depending on TGFB treatment. (III) Quantification of nuclear pJNK. (d) Immunofluorescence microscopy of primary fibroblasts of a healthy control individual treated with either DMSO, JNK Inh VIII, anisomycin (AM). (I) Endogenous MAPKBP1 stained with anti-MAPKBP1 antibody (MAPKBP1: red, acetylated α-tubulin: green, nuclei: blue). Scale bars equal 2 μm. (II) Quantification of MAPKBP1 intensity at the basal body. (III) Ciliary length. Black bordered white dots represent the mean of each independent experiment. Violin plots represent the sum of all individual datapoints. (e) Immunofluorescence microscopy of endogenous MAPKBP1 at the basal body of primary cilia in primary fibroblasts from a healthy control individual and a NPHP20 patient. (I) Endogenous MAPKBP1 was stained with primary anti-MAPKBP1 antibody (MAPKBP1: red, acetylated α-tubulin: green, nuclei: blue). Scale bars equal 2 μm. (II) Quantification of pJNK intensities at the basal body in dermal fibroblasts from healthy control individuals I and II, and NPHP20 patients I, II, and V. (III) Quantification of nuclear pJNK in dermal fibroblasts of healthy control I and II and NPHP20 patients I, II, and V. (IV) Ciliary length of primary cells derived from healthy control individuals I and II and NPHP20 patients I, II, and V with and without treatment with 250 nM cytochalasin D. Data are mean ± SEM of the mean values of at least 3 independent experiments. All data represent the mean ± SEM of at least 3 independent experiments. AM, Anisomycin; DMSO, dimethyl sulfoxide; JNK Inh VIII/JI VIII, JNK inhibitor VIII; siRNA, small interfering RNA; TGFB, transforming growth factor beta; #, ##, reference for statistical testing; ∗*P* ≤ 0.05; ∗∗*P* ≤ 0.01; ∗∗∗*P* ≤ 0.001; and ∗∗∗∗*P* ≤ 0.0001.

Finally, we aimed to investigate the JNK-MAPKBP1 axis on primary cilia in cells expressing endogenous MAPKBP1. Ciliated RPE1 cells exhibited a strong MAPKBP1 antibody staining at ciliary basal bodies (Figure 6aI and II; Supplementary Figure S11). Comparable to overexpression experiments, treatment with JI VIII and AM led to an increase and decrease of MAPKBP1 intensities, respectively (Figure 6aI and II; Supplementary Table S13). Furthermore, 1-hour treatment with the physiological JNK activator, transforming growth factor beta (TGFB) recapitulated findings observed with AM (Figure 6aI and II; Supplementary Figures S11 and S12; Supplementary Table S13). Loss of MAPKBP1 was accompanied by a significant length reduction of primary cilia upon both, AM and TGFB treatment (Figure 6aIII; Supplementary Table S13). To investigate whether ciliary length was directly related to MAPKBP1 loss from the ciliary base, MAPKBP1 was knocked down in RPE1 cells using siRNA (MAPKBP1-KD) (Figure 6bI and II; Supplementary Figure S13; Supplementary Table S14). MAPKBP1-KD cells showed a decrease in basal body localization of MAPKBP1 and a significantly reduced ciliary length (Figure 6bIII; Supplementary Table S14). Neither knockdown nor ciliary dissociation of MAPKBP1 with TGFB led to a change of pJNK intensity at basal bodies (Figure 6cI–II; Supplementary Figure S14; Supplementary Table S15). However, the amount of nuclear pJNK was significantly increased in MAPKBP1-KD cells independent of TGFB stimulation (Figure 6cIII; Supplementary Figure S14; Supplementary Table S15). Treatment of control fibroblasts with either JI VIII or AM recapitulated the findings in RPE1 cells (Figure 6dI–III; Supplementary Figure S15, Supplementary Table S16). However, in contrast to MAPKBP1-KD RPE1, pJNK at the basal body of primary fibroblasts was significantly reduced and nuclear pJNK intensity was comparable in patients and controls, suggestive of a regulatory rescue mechanism (Figure 6eI–III; Supplementary Figure S16; Supplementary Table S17). Recent investigations suggested an involvement of the actin cytoskeleton in JNK-dependent ciliogenesis regulation.^14^ In line with this report, treatment of patient cells with the actin depolymerizer, cytochalasin D allowed a full restoration of ciliary length (Figure 6eIV; Supplementary Table S18). In summary, ciliary localization of MAPKBP1 is strongly dependent on the activation status of JNK, directly determines ciliary length, and is implicated in the downstream regulation of intracellular pJNK trafficking.

## Discussion

With this study, we provide strong evidence for a reclassification of MAPKBP1-associated kidney disease into the spectrum of cilia-dependent NPH clinically characterized by a milder natural course. Unlike the initial study,^4^ we demonstrate endogenous MAPKBP1 to localize in a specific pattern to the ciliary basal body in primary fibroblasts from healthy individuals as well as RPE1 cells; and observed a small but significant decrease in ciliary length in patient-derived cells. However, unlike other basal body–associated ciliopathies, this condition presents with relatively mild and nonspecific clinical features, highlighting that only genetic testing can provide a definitive diagnosis. In our clinical end point analysis of chronic kidney disease progression, KF in MAPKBP1-associated disease showed a later onset—on average, about 10 years later—compared with all other forms of NPH.^1^^,^^2^ This is in line with the overall observation that pathogenic effects, including ciliary shortening appear to be relatively modest. Variable amounts of MAPKBP1 in healthy individuals highlight that even under regular conditions, MAPKBP1 basal body association is tightly regulated and impacts on ciliary length modulation. Thus, we assume that previous experiments accidently failed to detect ciliary MAPKBP1 and differences in ciliary length because of the choice or cultivation conditions of control cell lines. SiRNA-mediated knockdown of MAPKBP1 in RPE1 cells further demonstrated that MAPKBP1 loss directly induces ciliary length reduction.

Comparative overexpression of all known patient-related MAPKBP1 variants revealed a pronounced reduction in microtubular, centrosomal, and basal body localization; and conversely, a strong increase in diffuse cytosolic distribution (except for R1108C), generalizing previous results.^5^ Reduced ciliary frequency, in contrast to reduced ciliary length, detected in overexpression models could not be recapitulated in primary cells because of largely divergent growth rates and confluences. We speculate, however, that this effect could exert an impact in settings where protein amounts exceed endogenous expression levels. Despite their rather nonphysiological nature, overexpression experiments showed that although the CC-domain is necessary and sufficient for homodimerization of MAPKBP1, its 2 other protein domains, WD40 and JUD are similarly required for correct intracellular localization, providing an explanation for the pathogenicity of the 2 nontruncating variants. The rather wild type–like missense variant, R1108C located in the JBD corresponds to patient NPHP20_VI, a deceased baby, who was a homozygous carrier but most likely also affected by additional variations in other genes responsible for the severe phenotype.

**Figure 7:**
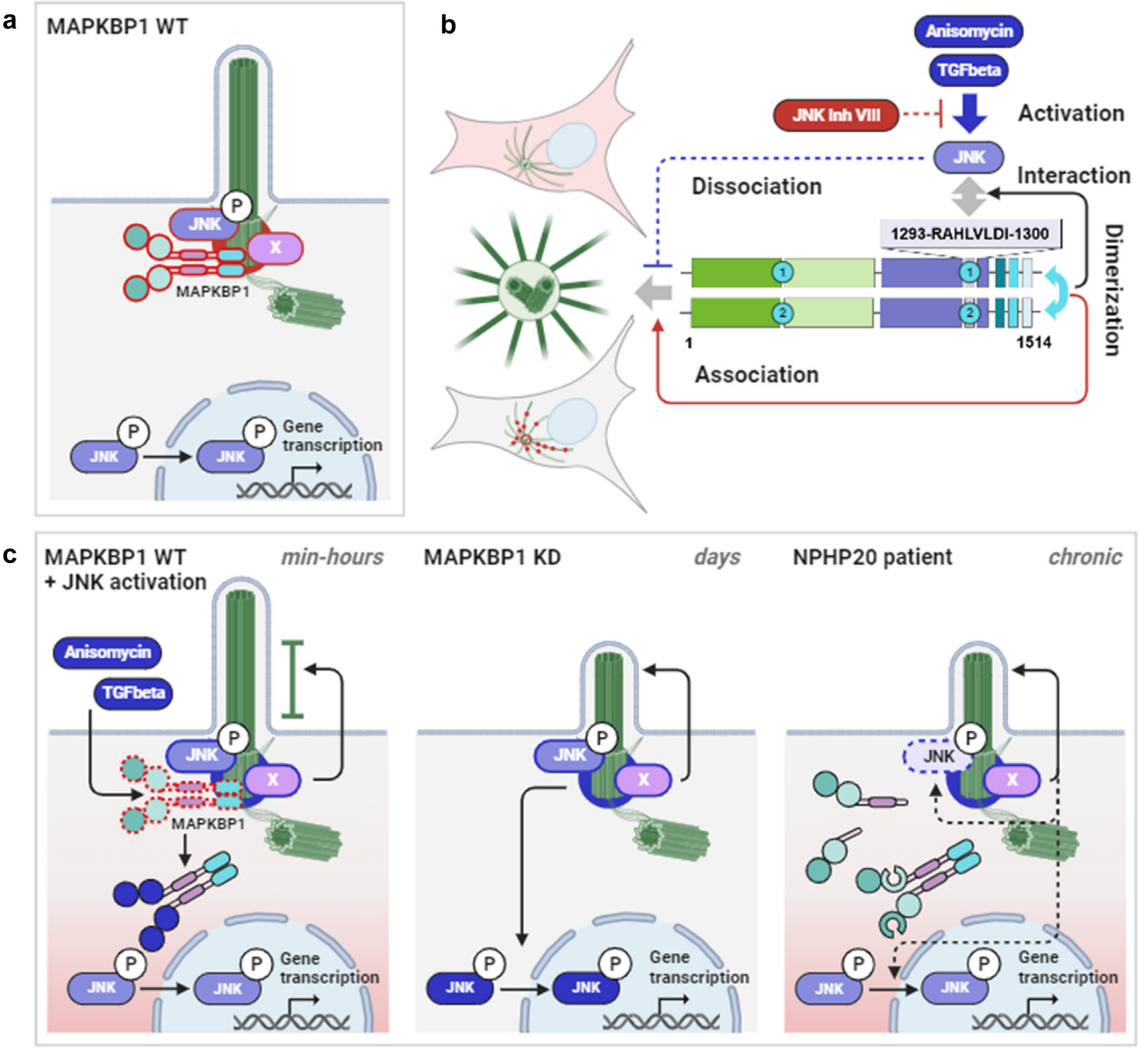
Hypothetical MAPKBP1-JNK-cilia-axis under physiological and pathophysiological conditions. (a) In healthy control cells, MAPKBP1 homodimers localize to the primary ciliary basal body. (b) Schematic representation of structure-activity relationships of MAPKBP1 domains: 2 CC-domains mediate homodimerization, which is a required to provide pairs of WD40 domains that allow association with microtubules, whereas binding of activated JNK by 2 D-Domains within the JBD is a prerequisite for dissociation from centrosomes and basal bodies. (c) JNK activation in healthy cells leads to a rapid loss of MAPKBP1 basal body association and accompanied by a reduction of ciliary length, which is eventually mediated by pJNK as well as unknown interaction partners at the basal body (“X”). Upon knock-down of MAPKBP1 for 2 to 3 days, increased nuclear translocation of pJNK but no change in basal body pJNK is observed. In cells of patients with NPHP20, where MAPKBP1 loss from primary cilia is a chronic state, nuclear pJNK is comparable to control cells, whereas pJNK at basal body is decreased. WT, wild type; X, unknown interaction partners at the basal body.

Because MAPKBP1 lacking its JBD showed the strongest loss of microtubular, centrosomal, and basal body association, and JBD was demonstrated to include the JNK-binding D-domain sequence, we hypothesize that JNK determines the association of MAPKBP1 with centriolar and microtubular structures. In both, overexpression cell models and cells endogenously expressing MAPKBP1, inhibition or activation of JNK led to an increase or complete loss of association with microtubules, centrosomes and basal bodies, respectively. Interestingly, MAPKBP1 dimers lacking one of the 2 JBD or WD40 domains exhibited an increased or decreased association to microtubular structures, respectively. Thus, we hypothesize that dimerization is a requirement for MAPKBP1 function by providing pairs of protein domains, which is a precondition for initial microtubular binding and JNK activation–dependent dissociation. It is tempting to speculate that the JBD-JNK interaction acts as a molecular switch between cytoskeleton bound and unbound states of MAPKBP1, which regulate ciliary length as a response to endogenous or exogenous stress signals (Figure 7b). Consequently, TGFB treatment appeared to be a physiological modulator of MAPKBP1 basal body localization and induced a rapid decrease in ciliary length even after 1-hour treatment (Figure 7c). TGFB-induced ciliary length reduction was previously only shown in osteoblasts, chondrocytes, and tenocytes.^15^, ^16^, ^17^, ^18^ Although speculative at the moment, ciliary length reduction in these cell types may refer to the spectrum of observed skeletal malformations in MAPKBP1-associated disease.

This study, adds additional evidence for a more generalizable effect of JNK-modulating molecules on primary ciliary phenotypes, which could be, at least be in part, regulated by MAPKBP1. Our findings suggest that JNK activation acts primarily upstream of MAPKBP1's functions involving so far unidentified interaction partners at the basal body, resulting in a direct disassembly of ciliary axonemes after dissociation. Thus, MAPKBP1 defects induce basal body conditions that are comparable to continuous JNK activation, which is in line with the inflammatory fibrosis observed in chronic tubulointerstitial nephritis of NPH. Interestingly, loss of CEP164/NPHP15, which shows a similar pattern at the ciliary base, was reported to increase epithelial to mesenchymal transition, a hallmark of renal fibrosis.^19^ Investigation of potential downstream effects of MAPKBP1 on JNK localization revealed an increased nuclear translocation of active pJNK in MAPKBP1-KD cells, which was independent of TGFB treatment and observed despite preserved basal body localization. Contrarily, in NPH patient-derived cells, pJNK was decreased at primary cilia, whereas nuclear pJNK intensities were similar to healthy controls. We assume that increased nuclear translocation of pJNK after siRNA-mediated KD represents an immediate response to an acute loss of MAPKBP1, which occurs independent of and later than ciliary shortening (Figure 7c). In contrast, we speculate that under sustained conditions of reduced ciliary MAPKBP1, the cell might adapt by activating rescue mechanisms to reduce both pJNK nuclear translocation and ciliary pJNK accumulation.

In line with our findings, pJNK was also localized to the base of motile and primary cilia in 2 very recent studies.^14^^,^^20^ Knock-down as well as JNK inhibition was found to impair primary ciliogenesis, resulting in a lower number of ciliated cells. Disturbed interactions between ciliary basal bodies and intraflagellar transport proteins and the actin cytoskeleton were proposed as molecular mechanisms following JNK signaling disruption.^14^ Interestingly, treatment with the actin depolymerization and ciliogenesis inducing agent cytochalasin D^21^ led to a complete restoration of ciliary length in cells of patients with primary NPHP20. Thus, MAPKBP1 might impact on ciliary phenotypes through JNK-dependent actin mechanisms, which, however remains speculative without further investigation. Elucidation of detailed molecular interactions with the actin cytoskeleton and the involved binding partners of pJNK and MAPKBP1 as well as investigation of the JNK signaling pathway in the pathophysiology of other NPHPs could lead to the identification of promising targets for the development of specific treatment strategies for patients with NPH.

## Disclosure

All the authors declared no competing interests.

## Supplementary Material

Supplementary File (PDF) **Supplemental Methods. Supplementary References. Figure S1.** Newly identified patient with NPHP20. **Figure S2.** Phenotypic comparison of patients with NPHP20 with aggregate data from the French NPH cohort. **Figure S3.** Immunofluorescence microscopy showing antibody staining of MAPKBP1 at the basal body of primary cilia in dermal fibroblast from healthy control individuals and patients with NPHP20. **Figure S4.** Immunofluorescence microscopy of HeLa cells transiently transfected with GFP-MAPKBP1 wild type, patient, and artificial deletion variants. **Figure S5.** Immunofluorescence microscopy of HeLa cells transiently transfected with GFP-MAPKBP1 wild type, patient, and artificial deletion variants. **Figure S6.** Immunofluorescence microscopy of H69 cells transiently transfected with GFP-MAPKBP1 wild- ype, patient, and artificial deletion variants. **Figure S7.** Immunofluorescence microscopy of HeLa cells transiently transfected with GFP-MAPKBP1 wild-type or deletion variants and treated with Taxol or Nocodazol, respectively. **Figure S8.** Characterization of MAPKBP1-delD variant. **Figure S9.** Immunofluorescence microscopy showing overall (A) intracellular, (B) centrosomal, and (C) ciliary localization of GFP-tagged MAPKBP1 wild type in transiently transfected (A, B) HeLa or (C) H69 cells upon either inhibition (JNK Inh VIII) or activation (anisomycin, AM) of JNK signaling pathway. **Figure S10.** Immunofluorescence microscopy of HeLa cells transiently cotransfected with RFP-tagged MAPKBP1 wild type and GFP-tagged MAPKBP1 deletion variants. **Figure S11.** Immunofluorescence microscopy of RPE1 cells treated with either DMSO, JNK Inh VIII, anisomycin (AM), or TGFβ. **Figure S12.** Quantification of MAPKBP1 intensity at the basal body in (A) RPE1 cells or (B) control dermal fibroblasts upon treatment with TGFβ or TNFα. **Figure S13.** Immunofluorescence microscopy of RPE1 cells transfected with anti-MAPKBP1 siRNA (MAPKBP1-KD) or scrambled siRNA. **Figure S14.** Immunofluorescence microscopy of endogenous pJNK at the basal body of primary cilia and in the nuclei of RPE1 cells transfected with anti-MAPKBP1 siRNA (MAPKBP1-KD) or scrambled siRNA and treated with either DMSO or TGFβ. **Figure S15.** Immunofluorescence microscopy of primary fibroblasts of a healthy control individual treated with either DMSO, JNK Inh VIII, or anisomycin (AM). **Figure S16.** Immunofluorescence microscopy of endogenous pJNK at the basal body of primary cilia and in the nuclei in primary fibroblasts from a healthy control individual and a patient with NPHP20. **Table S1.** Antibodies used for immunofluorescence microscopy. **Table S2.** Antibodies used for western blot. **Table S3.** Known pathogenic MAPKBP1 variants found in patients with NPH. **Table S4.** MAPKBP1 intensities at the basal body and ciliary length in dermal fibroblast from healthy control individuals and patients with NPHP20. **Table S5.** Quantification of cellular localization patterns for GFP-labeled MAPKBP1 wild type and variants overexpressed in HeLa cells. **Table S6.** GFP intensities at the centrosome of HeLa cells and at the basal body of H69 cells transiently transfected with GFP-tagged MAPKBP1 variants. **Table S7.** Coimmunoprecipitation (Co-IP) of HA-tagged MAPKBP1 wild type and DDK-tagged deletion variants overexpressed in HEK293T cells. **Table S8.** Coimmunoprecipitation (Co-IP) of GFP-tagged JNK2 and DDK-tagged MAPKBP1 variants overexpressed in HEK293T cells. **Table S9.** Quantification of JNK2-intensity in the nucleus in relation to the cytosol depending on the cotransfected MAPKBP1 variant. **Table S10.** GFP intensities at the centrosome of HeLa cells and the basal body of H69 cells transiently transfected with GFP-MAPKBP-WT or delD upon either inhibition (JNK Inh VIII) or activation (anisomycin = AM) of JNK signaling pathway. **Table S11.** Quantification of MAPKBP1 wild type in cell lysates of HEK293T cells treated with either DMSO, JNK Inh VIII, or anisomycin (AM), respectively (normalized to the amount in cells treated with DMSO, set to 100%). **Table S12.** Immunofluorescence microscopy of HeLa cells transiently cotransfected with RFP-MAPKBP1 wild type and GFP-MAPKBP1 variants. **Table S13.** MAPKBP1 intensities at basal body and ciliary length of untransfected RPE1 cells upon either inhibition (JNK Inh VIII) or activation (anisomycin = AM/TGFβ) of JNK signaling pathway. **Table S14.** MAPKBP1 intensities and ciliary length of RPE1 cells transfected with either scrambled siRNA or anti-MAPKBP1 control siRNA. **Table S15.** pJNK intensities at basal body and in the nucleus of RPE1 cells transfected with either scrambled siRNA or anti-MAPKBP1 control siRNA treated with either DMSO or TGFβ, respectively. **Table S16.** MAPKBP1 intensities at basal body and ciliary length of healthy control dermal fibroblasts upon either inhibition (JNK Inh VIII) or activation (anisomycin = AM) of JNK signaling pathway. **Table S17.** pJNK intensities at the basal body and in the nucleus in dermal fibroblasts of healthy control individuals and patients with NPHP20. **Table S18.** Ciliary length of primary cells derived from healthy control individuals I and II and NPHP20 patients I, II, and V with and without treatment with 250 nM cytochalasin D. STROBE statement.

## Footnotes

Supplementary File (PDF)

**Supplemental Methods.**

**Supplementary References.**
**Figure S1.** Newly identified patient with NPHP20. **Figure S2.** Phenotypic comparison of patients with NPHP20 with aggregate data from the French NPH cohort. **Figure S3.** Immunofluorescence microscopy showing antibody staining of MAPKBP1 at the basal body of primary cilia in dermal fibroblast from healthy control individuals and patients with NPHP20. **Figure S4.** Immunofluorescence microscopy of HeLa cells transiently transfected with GFP-MAPKBP1 wild type, patient, and artificial deletion variants. **Figure S5.** Immunofluorescence microscopy of HeLa cells transiently transfected with GFP-MAPKBP1 wild type, patient, and artificial deletion variants. **Figure S6.** Immunofluorescence microscopy of H69 cells transiently transfected with GFP-MAPKBP1 wild- ype, patient, and artificial deletion variants. **Figure S7.** Immunofluorescence microscopy of HeLa cells transiently transfected with GFP-MAPKBP1 wild-type or deletion variants and treated with Taxol or Nocodazol, respectively. **Figure S8.** Characterization of MAPKBP1-delD variant. **Figure S9.** Immunofluorescence microscopy showing overall (A) intracellular, (B) centrosomal, and (C) ciliary localization of GFP-tagged MAPKBP1 wild type in transiently transfected (A, B) HeLa or (C) H69 cells upon either inhibition (JNK Inh VIII) or activation (anisomycin, AM) of JNK signaling pathway. **Figure S10.** Immunofluorescence microscopy of HeLa cells transiently cotransfected with RFP-tagged MAPKBP1 wild type and GFP-tagged MAPKBP1 deletion variants. **Figure S11.** Immunofluorescence microscopy of RPE1 cells treated with either DMSO, JNK Inh VIII, anisomycin (AM), or TGFβ. **Figure S12.** Quantification of MAPKBP1 intensity at the basal body in (A) RPE1 cells or (B) control dermal fibroblasts upon treatment with TGFβ or TNFα. **Figure S13.** Immunofluorescence microscopy of RPE1 cells transfected with anti-*MAPKBP1* siRNA (MAPKBP1-KD) or scrambled siRNA. **Figure S14.** Immunofluorescence microscopy of endogenous pJNK at the basal body of primary cilia and in the nuclei of RPE1 cells transfected with anti-*MAPKBP1* siRNA (MAPKBP1-KD) or scrambled siRNA and treated with either DMSO or TGFβ. **Figure S15.** Immunofluorescence microscopy of primary fibroblasts of a healthy control individual treated with either DMSO, JNK Inh VIII, or anisomycin (AM). **Figure S16.** Immunofluorescence microscopy of endogenous pJNK at the basal body of primary cilia and in the nuclei in primary fibroblasts from a healthy control individual and a patient with NPHP20. **Table S1.** Antibodies used for immunofluorescence microscopy. **Table S2.** Antibodies used for western blot. **Table S3.** Known pathogenic MAPKBP1 variants found in patients with NPH. **Table S4.** MAPKBP1 intensities at the basal body and ciliary length in dermal fibroblast from healthy control individuals and patients with NPHP20. **Table S5.** Quantification of cellular localization patterns for GFP-labeled MAPKBP1 wild type and variants overexpressed in HeLa cells. **Table S6.** GFP intensities at the centrosome of HeLa cells and at the basal body of H69 cells transiently transfected with GFP-tagged MAPKBP1 variants. **Table S7.** Coimmunoprecipitation (Co-IP) of HA-tagged MAPKBP1 wild type and DDK-tagged deletion variants overexpressed in HEK293T cells. **Table S8.** Coimmunoprecipitation (Co-IP) of GFP-tagged JNK2 and DDK-tagged MAPKBP1 variants overexpressed in HEK293T cells. **Table S9.** Quantification of JNK2-intensity in the nucleus in relation to the cytosol depending on the cotransfected MAPKBP1 variant. **Table S10.** GFP intensities at the centrosome of HeLa cells and the basal body of H69 cells transiently transfected with GFP-MAPKBP-WT or delD upon either inhibition (JNK Inh VIII) or activation (anisomycin = AM) of JNK signaling pathway. **Table S11.** Quantification of MAPKBP1 wild type in cell lysates of HEK293T cells treated with either DMSO, JNK Inh VIII, or anisomycin (AM), respectively (normalized to the amount in cells treated with DMSO, set to 100%). **Table S12.** Immunofluorescence microscopy of HeLa cells transiently cotransfected with RFP-MAPKBP1 wild type and GFP-MAPKBP1 variants. **Table S13.** MAPKBP1 intensities at basal body and ciliary length of untransfected RPE1 cells upon either inhibition (JNK Inh VIII) or activation (anisomycin = AM/TGFβ) of JNK signaling pathway. **Table S14.** MAPKBP1 intensities and ciliary length of RPE1 cells transfected with either scrambled siRNA or anti-*MAPKBP1* control siRNA. **Table S15.** pJNK intensities at basal body and in the nucleus of RPE1 cells transfected with either scrambled siRNA or anti-*MAPKBP1* control siRNA treated with either DMSO or TGFβ, respectively. **Table S16.** MAPKBP1 intensities at basal body and ciliary length of healthy control dermal fibroblasts upon either inhibition (JNK Inh VIII) or activation (anisomycin = AM) of JNK signaling pathway. **Table S17.** pJNK intensities at the basal body and in the nucleus in dermal fibroblasts of healthy control individuals and patients with NPHP20. **Table S18.** Ciliary length of primary cells derived from healthy control individuals I and II and NPHP20 patients I, II, and V with and without treatment with 250 nM cytochalasin D. STROBE statement.
