# Supplementary material for: Structure-Activity Analysis Reveals Perturbed Cilia-Jun N-Terminal Kinase Signaling in MAPKBP1-Associated Kidney Disease
Source: Kidney Int Rep. 2025 Jun 4;10(8):2836–51. doi: 10.1016/j.ekir.2025.05.049 (PMC12347751; doi:10.1016/j.ekir.2025.05.049)
Supplement: Supplementary File (PDF) — Supplemental Methods. Supplementary References. Figure S1. Newly identified patient with NPHP20. Figure S2. Phenotypic comparison of patients with NPHP20 with aggregate data from the French NPH cohort. Figure S3. Immunofluorescence microscopy showing antibody staining of MAPKBP1 at the basal body of primary cilia in dermal fibroblast from healthy control individuals and patients with NPHP20. Figure S4. Immunofluorescence microscopy of HeLa cells transiently transfected with GFP-MAPKBP1 wild type, patient, and artificial deletion variants. Figure S5. Immunofluorescence microscopy of HeLa cells transiently transfected with GFP-MAPKBP1 wild type, patient, and artificial deletion variants. Figure S6. Immunofluorescence microscopy of H69 cells transiently transfected with GFP-MAPKBP1 wild- ype, patient, and artificial deletion variants. Figure S7. Immunofluorescence microscopy of HeLa cells transiently transfected with GFP-MAPKBP1 wild-type or deletion variants and treated with Taxol or Nocodazol, respectively. Figure S8. Characterization of MAPKBP1-delD variant. Figure S9. Immunofluorescence microscopy showing overall (A) intracellular, (B) centrosomal, and (C) ciliary localization of GFP-tagged MAPKBP1 wild type in transiently transfected (A, B) HeLa or (C) H69 cells upon either inhibition (JNK Inh VIII) or activation (anisomycin, AM) of JNK signaling pathway. Figure S10. Immunofluorescence microscopy of HeLa cells transiently cotransfected with RFP-tagged MAPKBP1 wild type and GFP-tagged MAPKBP1 deletion variants. Figure S11. Immunofluorescence microscopy of RPE1 cells treated with either DMSO, JNK Inh VIII, anisomycin (AM), or TGFβ. Figure S12. Quantification of MAPKBP1 intensity at the basal body in (A) RPE1 cells or (B) control dermal fibroblasts upon treatment with TGFβ or TNFα. Figure S13. Immunofluorescence microscopy of RPE1 cells transfected with anti-MAPKBP1 siRNA (MAPKBP1-KD) or scrambled siRNA. Figure S14. Immunofluorescence microscopy of endogenous pJNK at [file mmc1.pdf]

Supplemental Methods

General cell culture

HEK293T (DMSZ, Braunschweig, Germany) and HeLa (ATCC- LGC Standards, Manassas, USA) cells were cultivated in DMEM (Gibco, Thermo Fisher Scientific, Waltham, MA, USA) with 10% FBS (Biochrom GmbH, Berlin, Germany). H69 cholangiocytes [22] and RPE1 cells [4] were cultivated in DMEM/F-12 (Gibco, Thermo Fisher Scientific, Waltham, MA, USA) with 10% FBS, 1% MEM NEAA (Gibco, Thermo Fisher Scientific, Waltham, MA, USA) and 0.1% Penicillin-Streptomycin (Gibco, Thermo Fisher Scientific, Waltham, MA, USA). Dermal fibroblasts from individuals NPHP20_I, NPHP20_II and NPHP20_V were previously published [4]; fibroblasts were cultivated in DMEM supplemented with 10% FBS and 1% Penicillin-Streptomycin. All cells were maintained at 37°C and 5% CO_2 in humidified atmosphere.

Expression vectors

MAPKBP1 variants were prepared using an expression vector encoding N-terminally GFP-tagged wild type MAPKBP1 (pcDNA3.1D GFP_JNKBP1/2xDDK-His-TOPO) [5] and the Q5 site directed mutagenesis kit according to the manufacturer's protocol (New England Biolabs, Ipswich, USA). Expression vectors encoding N-terminally RFP-tagged or C-terminally HA- or DDK-tagged MAPKBP1 wild type were previously published [5]. GFP-JNK2 was a gift from Rony Seger (Addgene plasmid # 86831) [S1]. JNK2 with three N-terminally fused HA-tags was previously described [4].

Co-Immunoprecipitation (Co-IP) and Western Blot

HEK293T cells grown in 25 cm^2 cell culture flasks to 70% confluency were transiently co-transfected with Flag(DDK)-tagged MAPKBP1 variants and GFP-JNK2 using FuGene (Promega, Madison, USA). After 24 hours, cells were lysed in RIPA or IP-lysis buffer (Thermo Fisher Scientific, Waltham, USA) with protease and phosphatase Inhibitor cocktail (Sigma-Aldrich, St. Louis, USA). Co-IP was performed using Anti-FLAG® M2 Magnetic Beads or Pierce™ HA-Tag Magnetic IP/Co-IP Kit (Thermo Fisher Scientific, Waltham, USA) according to manufacturer's instructions. Samples were run on an 3-8% Tris-Acetate SDS gel and blotted on a PVDF membrane (Thermo Fisher Scientific, Waltham, USA) using the iBlot 2 dry blotting system. Primary and secondary antibodies (Table S2) were incubated at 4°C over night and at room temperature for one hour, respectively. The protein bands were detected using the SuperSignal™ West Femto (Thermo Fisher Scientific, Waltham, USA). Band intensities were quantified using ImageJ. Co-IP eluates were normalized to corresponding lysates to account for variable expression.

Table S1: Antibodies used for immunofluorescence microscopy.

antibody	dilution	manufacturer
anti-Pericentrin (ab28144)	1:500	Abcam
anti-HA (TRITC) (sc-7392)	1:100	Santa Cruz
anti-ac alpha tubulin 2 (EPR16772)	1:200	Abcam
anti MAPKBP1 (HPA030832)	1:300	Sigma Aldrich
anti-ac alpha tubulin (6-11B-1)	1:200	Thermo Fisher
anti-alpha Tubulin (ab52866; clone EP1332Y)	1:400	Abcam
anti-Phospho.JNK1/2 (Thr183/Tyr185) (44-682G)	1:1500 - 1:100	Invitrogen
goat anti-mouse AF 568	1:500	Thermo Fisher
goat anti-rabbit AF488	1:500	Thermo Fisher
goat anti-rabbit AF 555	1:100	Thermo Fisher
goat anti-mouse AF488	1:300	Thermo Fisher

Table S2: Antibodies used for western blot

antibody	dilution	manufacturer
anti-SAPK/JNK	1:1000	Cell Signaling
anti-DDK (clone FG4R)	1:500	Thermo Fisher
anti-HA (clone F-7)	1:100	Santa Cruz
anti-rabbit IgG (HRP-linked)	1:2000	Cell Signaling
anti-mouse IgG (HRP linked)	1:5000	Cell Signaling

Table S3: Known pathogenic MAPKBP1 variants found in patients with NPH.

Individual # in this study	Zygosity	cDNA change	Protein change	Parental consanguinity	Age Publication	Age ESRD	Kidney Phenotype	Skeletal Phenotype	Additional information	Reference
NPHP20_I_I	Comp het	c.592C>T c.4393C>T	p.Arg198* p.Arg1465*	No	25	-	na	loose patella; long fingers and feet	na	Macia et al. (2017)
NPH20P_I_II	Comp het	c.592C>T c.4393C>T	p.Arg198* p.Arg1465*	No	27	-	na	statural growth delay	RP; CHD; severe meningomyelocele	Macia et al. (2017)
NPHP20_II	Comp het	c.1318C>T	p.Arg440* p.396_445del	Yes	na	12	KTx at 15	facial dysmorphism	na	Macia et al. (2017)
NPHP20_III	Hom	c.1631G>A	p.Arg544Gln	Yes	23	-	na	na	na	Macia et al. (2017)
NPHP20_IV	Hom	c.2444-1G>A	p.Leu814fs40*	Yes	na	15	na	short stature	na	Macia et al. (2017)
NPHP20_V_I	Hom	c.2827C>T	p.Gln943*	Yes	na	22	KTx at 22+39	scoliosis	na	Macia et al. (2017)
NPHP20_V_II	Hom	c.2827C>T	p.Gln943*	Yes	na	25	KTx at 27+38; vesicoureteral reflux	scoliosis	cholesteatoma; amyloid angiopathy	Macia et al. (2017)
NPHP20_V_III	Hom	c.2827C>T	p.Gln943*	Yes	na	20	KTx at 21	Scoliosis; short stature; palate cleft	mild mental retardation	Macia et al. (2017)
NPHP20_VI	Hom	c.3322C>T	p.Arg1108Cys #	na	na	-	deceased baby	na	na	Shamseldin et al. (2020)
NPHP20_VII	Hom	c.4317+5G>C	p.Gly1442Valfs*12	Yes	37	23	KTx at age 23 y; increased echogenicity; solitary cysts (left and right kidney)	facial dysmorphism with dental overcrowding; scoliosis; ED-like syndrome;	mild exophthalmos	Schönauer et al. (2020)
NPHP20_VIII	Hom	c.3333-2A>G	p.Glu1112Tyrfs*41 p.Arg1111Serfs*5 p.Pro1113Glufs*2	No	14	-	CKD G3b at age 14 y; polyuria; polydipsia; increased echogenicity; solitary cysts (left and right kidney)	growth retardation	vascular anomaly: left IVC	Schönauer et al. (2020)
NPHP20_IX_I	Hom	c.952C>T	p.Arg318*	Yes	31	30	CKD at age 19; renal ultrasound in 2015 showed echogenic kidneys with multiple bilateral cysts	na	na	Al-Hamed M. (2021)
NPHP20_IX_II	Hom	c.952C>T	p.Arg318*	Yes	12	-	NPH diagnosis by renal biopsy	na	na	Al-Hamed M. (2021)
NPHP20_X	Hom	c.2493_2497del	p.Ser832Glyfs*34	na	20	17	Small echogenic kidney with multiple cysts; CKD stage V	Scoliosis	weight under the normal percentile for age; bartter syndrome; hyperlipidemia; Gallstones; high PTH	Unpublished/this study
NPHP20_XI	Comp het	c.2620C>T c.3332+1G>A	p.Gln874* p.?	na	12	-	CKD stage 2; renal cysts; high echogenicity; abnormal corticomedullary differentiation; glomerulosclerosis; tubulointerstitial damage	na	na	Sakakibara et al. (2022)
NPHP20_XII	Hom	c.2271del	p.Gly758Aspfs*74	na	20	20	eGFR = 9	na	na	Nigro et al. (2023)

CHD: coronary heart disease; CKD: chronic kidney disease; ESRD: end stage renal disease; IVC: inferior vena cava; KTx: kidney transplantation; RP: retinitis pigmentosa; PTH: parathyroid hormone; # = variant of unknown significance

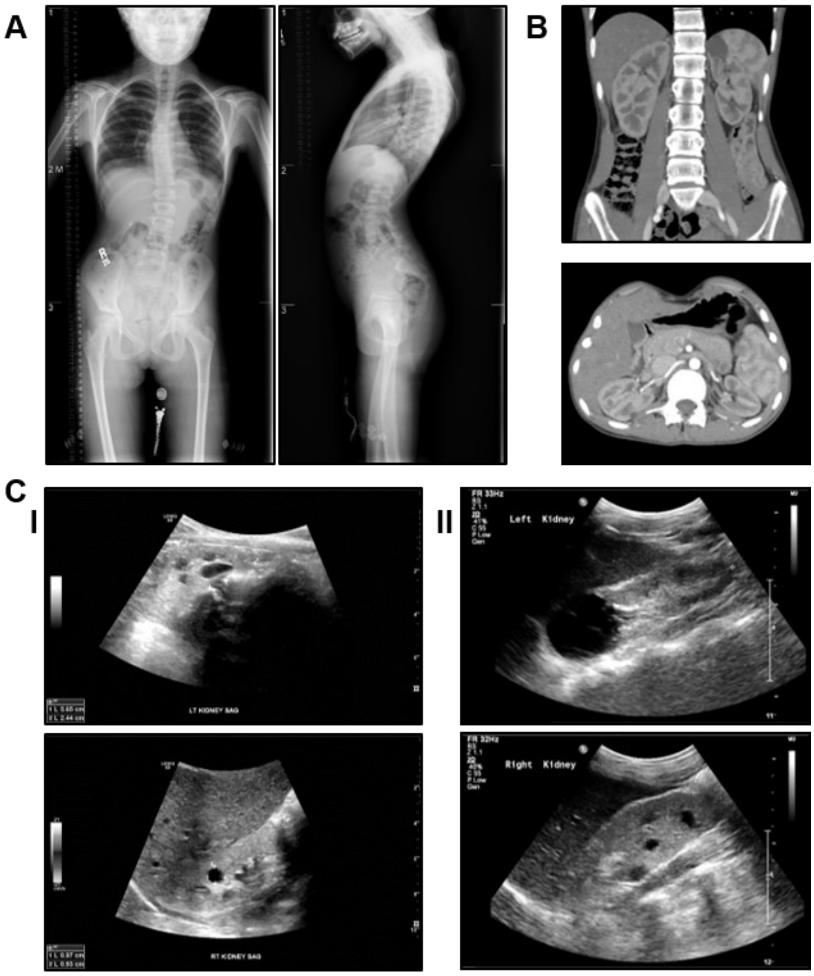

Figure S1: Newly identified NPHP20 patient. **(A)** X-ray images of NPHP20_X showing severe scoliosis. **(B)** CT scan of NPHP20_IX_I. **(C)** Renal ultrasound of (I) NPHP20_X and (II) NPHP20_IX_I. Upper panels: left kidney; lower panels: right kidney.

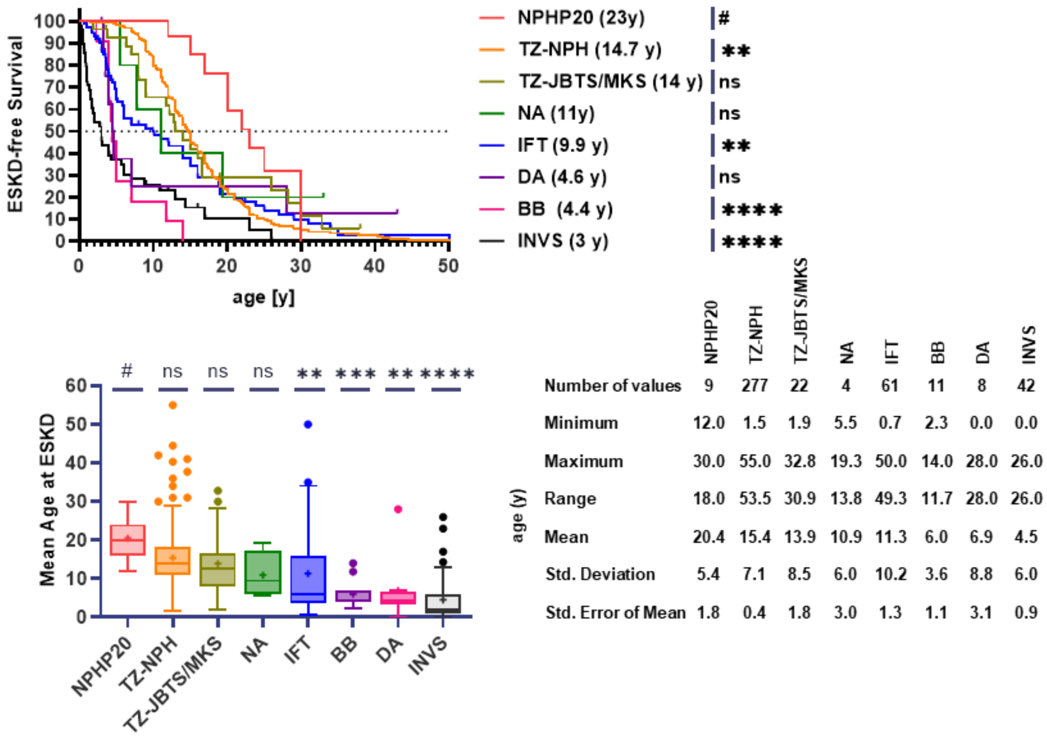

	NPHP20	TZ-NPH	TZ-JBTS/MKS	NA	IFT	BB	DA	INVS
Number of values	9	277	22	4	61	11	8	42
Minimum	12.0	1.5	1.9	5.5	0.7	2.3	0.0	0.0
Maximum	30.0	55.0	32.8	19.3	50.0	14.0	28.0	26.0
Range	18.0	53.5	30.9	13.8	49.3	11.7	28.0	26.0
Mean	20.4	15.4	13.9	10.9	11.3	6.0	6.9	4.5
Std. Deviation	5.4	7.1	8.5	6.0	10.2	3.6	8.8	6.0
Std. Error of Mean	1.8	0.4	1.8	3.0	1.3	1.1	3.1	0.9

Figure S2: Phenotypic comparison of NPHP20 patients with aggregate data from the french NPH cohort [2]. Genes associated with certain ciliary modules were combined into distinct subgroups: BB: basal body, DA: distal appendage, IFT: intraflaggelar transport complex, INVS: inversin compartment, NA: no clear association, TZ-JBTS/MKS: transition zone-Joubert syndrome/Meckel-Gruber syndrome, TZ-NPH: transition zone-Nephronophthisis. Upper panel: Kaplan-Meier survival analysis, lower panel: mean age at ESKD.

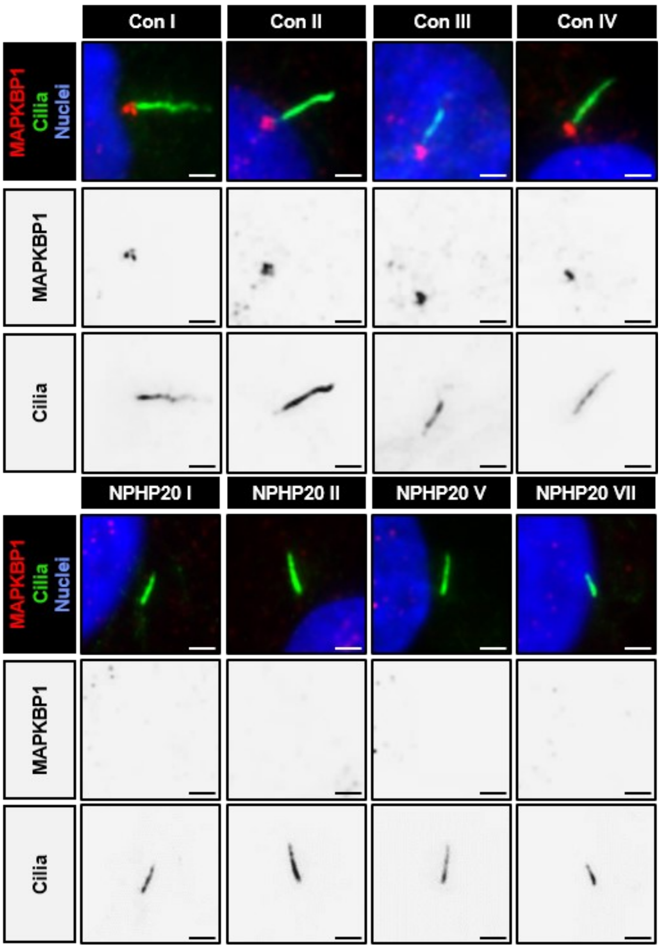

Figure S3: Immunofluorescence microscopy showing antibody staining of MAPKBP1 at the basal body of primary cilia in dermal fibroblast from healthy control individuals and NPHP20 patients (MAPKBP1: red, acetylated α-tubulin: green, nuclei: blue). Scale bars equal 2 µm.

Table S4: MAPKBP1 intensities at the basal body and ciliary length in dermal fibroblast from healthy control individuals and NPHP20 patients. Data are mean±SEM. n=number of independent experiments (minimum cell number measured in each experiment)

variant	intensity at basal body<br>[x-fold cytosol; cytosol Con I set to 1]	ciliary length<br>[µm]
Con I	6.6±0.9 n=5 (20)	4.7±0.1 n=8 (66)
Con II	5.5±0.5 n=5 (6)	4.6±0.1 n=5 (46)
Con III	2.9±0.7 n=3 (20)	3.7±0.1 n=6 (28)
Con IV	5.7±0.5 n=3 (30)	4.6±0.2 n=3 (54)
NPHP20 I	1.0±0.1 n=3 (19)	3.8±0.2 n=3 (101)
NPHP20 II	1.4±0.2 n=3 (20)	4.1±0.02 n=3)101)
NPHP20 V	1.1±0.1 n=3 (20)	4.2±0.1 n=4 (100)
NPHP20 VII	1.3±0.1 n=3 (20)	2.1±0.1 n=4 (102)

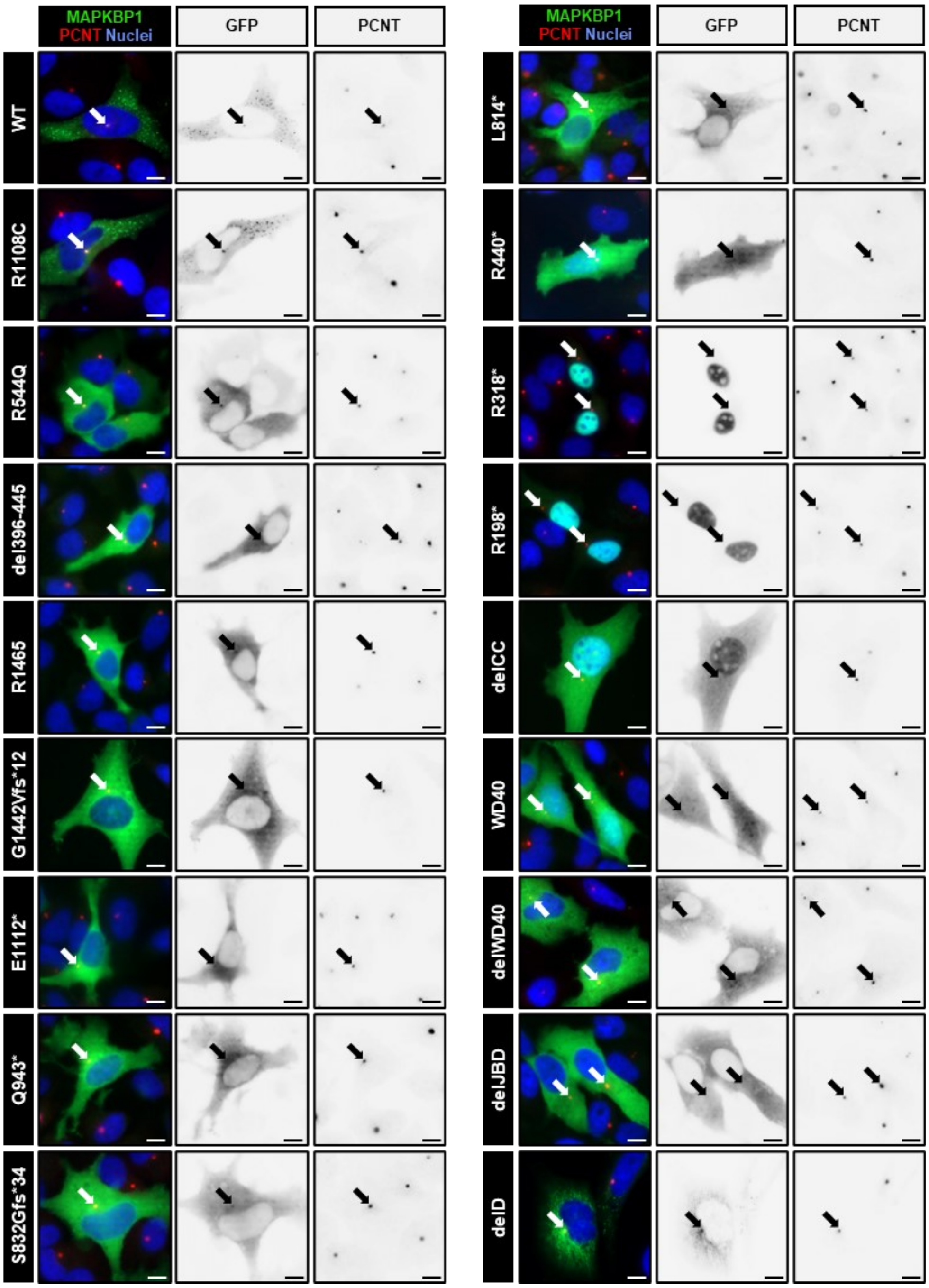

Figure S4: Immunofluorescence microscopy of HeLa cells transiently transfected with GFP-MAPKBP1 wild-type, patient and artificial deletion variants. Centrosomes were stained with anti-pericentrin (PCNT) antibody (GFP-MAPKBP1: green, PCNT: red, nuclei: blue). Representative example images are shown for the overall subcellular localization. Scale bars equal 10 µm.

Table S5: Quantification of cellular localization patterns for GFP-labeled MAPKBP1 wild-type and variants overexpressed in HeLa cells. Data are mean±SEM. n=number of independent experiments (minimum cell number counted in each experiment)

variant	centrosomal [% transfected cells]	punctate [% transfected cells]	filamentous [% transfected cells]	cytosolic [% transfected cells]	nuclear [% transfected cells]
WT	98.8±0.4 n=10 (37)	75.2±2.9 n=10 (37)	2.8±0.9 n=10 (37)	1.4±0.6 n=10 (37)	0.0±0.0 n=10 (37)
R1108C	98.3±1.1 n=3 (54)	85.3±6.5 n=3 (54)	0.0±0.0 n=3 (54)	2.6±2.6 n=3 (54)	0.0±0.0 n=3 (54)
R544Q	91.9±3.1 n=3 (43)	43.8±5.7 n=3 (43)	0.0±0.0 n=3 (43)	51.1±3.5 n=3 (43)	0.6±0.6 n=3 (43)
del396-445	0.0±0.0 n=3 (50)	0.0±0.0 n=3 (50)	0.0±0.0 n=3 (50)	100.0±0.0 n=3 (50)	0.0±0.0 n=3 (50)
R1465*	39.2±8.3 n=3 (46)	2.6±1.3 n=3 (46)	0.0±0.0 n=3 (46)	100.0±0.0 n=3 (46)	14.9±4.3 n=3 (46)
G1442Vfs*12	31.8±8.4 n=3 (29)	6.3±3.5 n=3 (29)	0.0±0.0 n=3 (29)	100.0±0.0 n=3 (29)	22.5±12.7 n=3 (29)
E1112*	8.9±4.5 n=3 (46)	0.0±0.0 n=3 (46)	0.0±0.0 n=3 (46)	100.0±0.0 n=3 (46)	17.7±4.6 n=3 (46)
Q943*	31.9±2.1 n=3 (43)	0.0±0.0 n=3 (43)	0.0±0.0 n=3 (43)	100.0±0.0 n=3 (43)	37.7±14.5 n=3 (43)
S832Gfs*34	50.1±5.2 n=3 (55)	0.0±0.0 n=3 (55)	0.0±0.0 n=3 (55)	100.0±0.0 n=3 (55)	24.5±4.8 n=3 (55)
L814*	18.8±4.4 n=3 (24)	0.0±0.0 n=3 (24)	0.0±0.0 n=3 (24)	100.0±0.0 n=3 (24)	26.6±5.1 n=3 (24)
R440*	40.4±6.2 n=3 (44)	2.3±2.3 n=3 (44)	0.0±0.0 n=3 (44)	100.0±0.0 n=3 (44)	99.2±0.8 n=3 (44)
R318*	0.0±0.0 n=3 (67)	0.0±0.0 n=3 (67)	0.0±0.0 n=3 (67)	7.4±2.2 n=3 (67)	100.0±0.0 n=3 (67)
R198*	0.0±0.0 n=3 (52)	0.0±0.0 n=3 (52)	0.0±0.0 n=3 (52)	0.9±0.9 n=3 (52)	100.0±0.0 n=3 (52)
delCC	44.1±2.0 n=4 (30)	4.9±1.4 n=4 (30)	0.0±0.0 n=4 (30)	100.0±0.0 n=4 (30)	77.1±9.2 n=4 (30)
WD40	40.6±4.2 n=4 (61)	1.9±1.0 n=4 (61)	0.0±0.0 n=4 (61)	100.0±0.0 n=4 (61)	83.4±6.1 n=4 (61)
delWD40	28.7±7.8 n=4 (50)	13.0±4.6 n=4 (50)	0.0±0.0 n=4 (50)	99.2±0.5 n=4 (50)	18.2±4.3 n=4 (50)
delJBD	6.6±2.6 n=4 (49)	5.5±1.8 n=4 (49)	0.0±0.0 n=4 (49)	97.1±1.7 n=4 (49)	4.5±1.3 n=4 (49)
delD	97.3±1.5 n=4 (32)	63.2±8.0 n=4 (32)	6.4±1.6 n=4 (32)	2.8±1.1 n=4 (32)	0.0±0.0 n=4 (32)

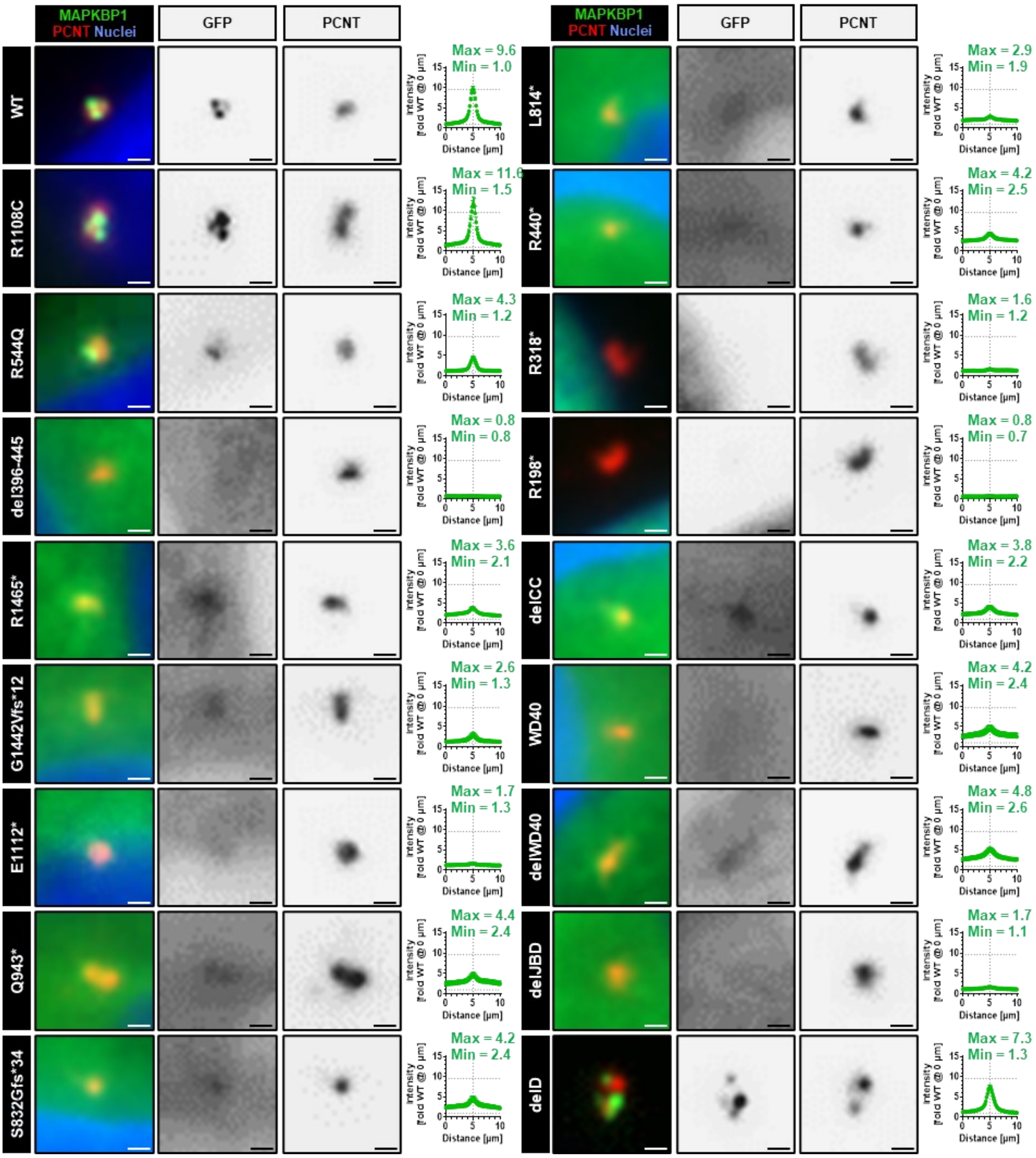

Figure S5: Immunofluorescence microscopy of HeLa cells transiently transfected with GFP-MAPKBP1 wild-type, patient and artificial deletion variants. Centrosomes were stained with anti-pericentrin (PCNT) antibody (GFP-MAPKBP1: green, PCNT: red, nuclei: blue). Representative example images are shown for the centrosomal localization. Scale bars equal 1 µm. Quantification of GFP intensity at the centrosome relative to the cytosol of HeLa cells transiently transfected with GFP-MAPKBP1 variants. Data are mean ± SEM of at least three independent experiments normalized to GFP intensity of MAPKBP1-WT in the cytosol (set to 1).

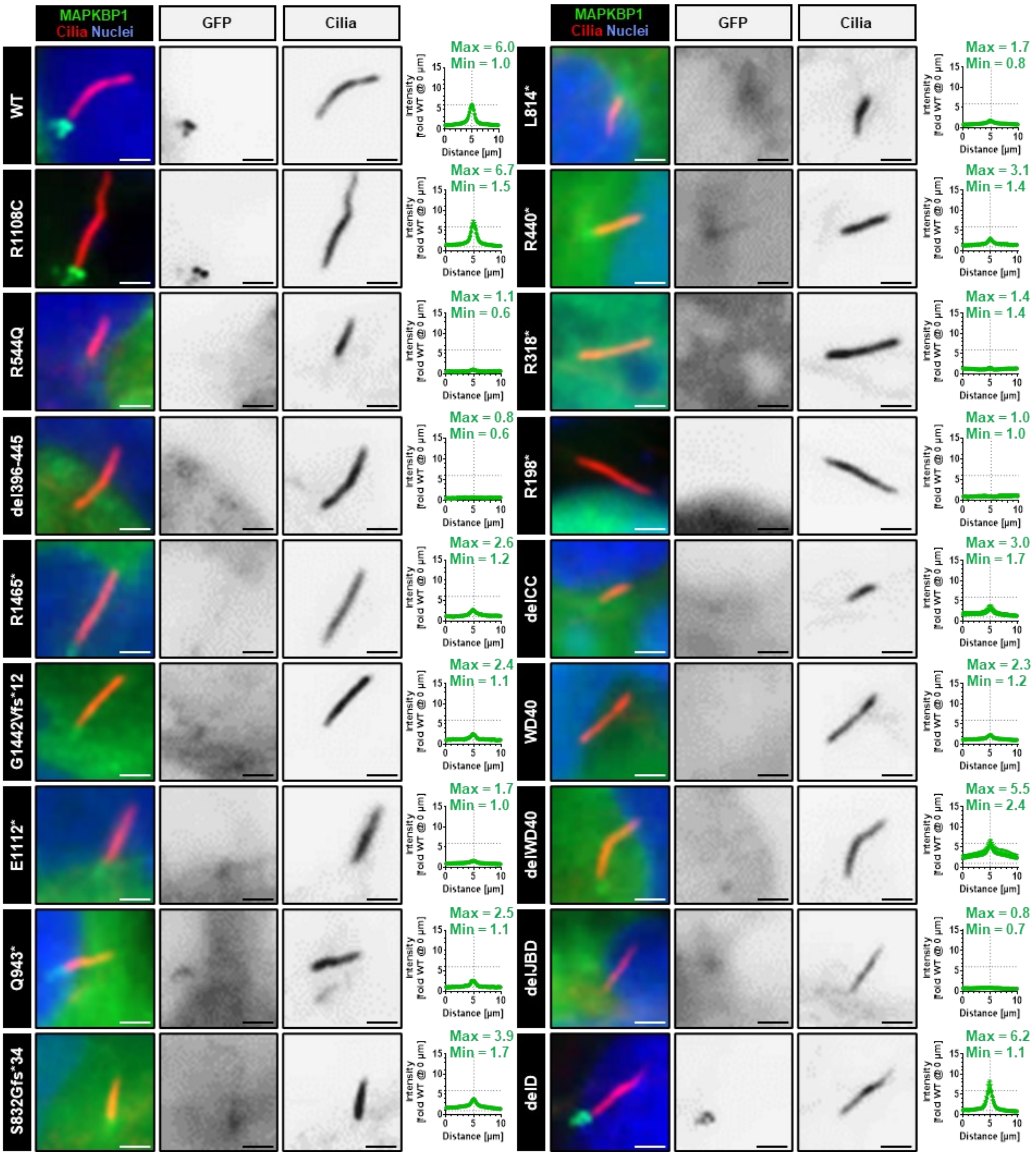

Figure S6: Immunofluorescence microscopy of H69 cells transiently transfected with GFP-MAPKBP1 wild-type, patient and artificial deletion variants. Primary cilia were stained with anti-acetylated α-tubulin antibody (GFP-MAPKBP1: green, acetylated α-tubulin: red, nuclei: blue). Representative example images are shown for the ciliary localization. Scale bars equal 2 µm. Quantification of GFP intensity at the centrosome relative to the cytosol of HeLa cells transiently transfected with GFP-MAPKBP1 variants. Data are mean ± SEM of at least three independent experiments normalized to GFP intensity of MAPKBP1-WT in the cytosol (set to 1).

Table S6: GFP intensities at the centrosome of HeLa cells and at the basal body of H69 cells transiently transfected with GFP-tagged MAPKBP1 variants. Data are mean±SEM. n=number of independent experiments (minimum cell number measured in each experiment)

variant	intensity at centrosome [x-fold cytosol; cytosol WT set to 1]	intensity at basal body [x-fold cytosol; cytosol WT set to 1]	ciliary length [µm]	cilium present [% transfected cells]	localization at basal body [% transfected cells]
WT	9.6±0.8 n=16 (28)	6.0±0.4 n=12 (11)	5.4±0.1 n=12 (11)	8.9±1.3 n=9 (120)	8.3±1.3 n=9 (6)
R1108C	8.0±1.0 n=3 (50)	4.4±0.1 n=3 (40)	5.5±0.1 n=3 (38)	8.6±0.3 n=3 (856)	8.2±0.4 n=3 (74)
R544Q	3.7±0.4 n=3 (50)	1.8±0.4 n=3 (6)	5.1±0.1 n=3 (6)	2.0±0.2 n=3 (108)	0.7±0.2 n=3 (6)
del396-445	1.1±0.1 n=3 (50)	1.4±0.3 n=3 (5)	4.8±0.8 n=3 (5)	2.6±0.5 n=3 (104)	0.0±0.0 n=3 (5)
R1465*	1.8±0.02 n=3 (50)	2.3±0.4 n=3 (13)	4.1±0.4 n=3 (13)	3.5±0.3 n=3 (117)	1.8±0.8 n=3 (14)
G1442Vfs*12	1.8±0.3 n=3 (30)	2.4±0.4 n=3 (8)	4.1±0.2 n=3 (8)	3.3±1.0 n=3 (103)	1.6±0.7 n=3 (8)
E1112*	1.3±0.1 n=3 (44)	1.8±0.1 n=3 (22)	4.5±0.1 n=3 (22)	6.9±0.4 n=3 (106)	2.1±0.9 n=3 (7)
Q943*	1.9±0.2 n=3 (50)	2.3±0.3 n=3 (6)	3.7±0.4 n=3 (7)	2.8±0.9 n=3 (122)	1.8±0.9 n=3 (7)
S832Gfs*34	1.7±0.1 n=3 (50)	2.3±0.1 n=3 (32)	4.5±0.1 n=3 (31)	5.0±0.4 n=3 (854)	3.3±0.3 n=3 (39)
L814*	1.5±0.1 n=3 (25)	2.1±0.1 n=3 (20)	3.9±0.1 n=3 (21)	7.0±1.1 n=3 (85)	1.3±0.3 n=3 (6)
R440*	1.7±0.01 n=3 (50)	2.2±0.1 n=3 (18)	4.0±0.2 n=3 (18)	1.9±0.7 n=3 (108)	1.0±0.6 n=3 (18)
R318*	1.4±0.04 n=3 (50)	0.9±0.1 n=3 (41)	5.9±0.5 n=3 (40)	6.2±0.8 n=3 (860)	0.6±0.02 n=3 (47)
R198*	1.3±0.2 n=3 (50)	0.9±0.2 n=3 (5)	5.0±0.4 n=3 (5)	3.3±0.3 n=3 (111)	0.2±0.1 n=3 (3)
delCC	1.8±0.1 n=4 (50)	1.8±0.1 n=3 (28)	4.8±0.2 n=3 (28)	7.2±0.6 n=3 (876)	4.0±0.2 n=3 (53)
WD40	1.7±0.03 n=4 (50)	2.1±0.2 n=3 (26)	4.5±0.2 n=3 (26)	6.4±0.9 n=3 (782)	4.1±0.9 n=3 (38)
delWD40	1.9±0.1 n=4 (50)	2.4±0.1 n=3 (35)	4.7±0.3 n=3 (32)	5.4±0.7 n=3 (848)	3.9±0.1 n=3 (41)
delJBD	1.4±0.1 n=4 (50)	1.2±0.2 n=3 (14)	5.2±0.1 n=3 (14)	6.8±0.4 n=3 (773)	0.2±0.1 n=3 (47)
delD	6.0±0.9 n=4 (50)	5.5±0.8 n=3 (30)	5.4±0.2 n=3 (30)	10.8±0.3 n=3 (797)	10.0±0.2 n=3 (80)
untransfected			4.9±0.3 n=8 (19)		

Table S7: Co-immunoprecipitation (Co-IP) of HA-tagged MAPKBP1 wild-type and DDK-tagged deletion variants overexpressed in HEK293T cells. Analysis of homodimerization efficiency by quantification of western blot bands. Data are mean±SEM; WT/WT homodimerization was set to 1 in each individual experiment.

homodimerization	interaction with WT [x-fold WT/WT]
WT-DDK/WT-HA	1.0±0.0 n=3
delCC-DDK/WT-HA	0.1±0.1 n=3
WD40-DDK/WT-HA	0.0±0.0 n=3
delWD40-DDK/WT-HA	0.9±0.5 n=3
delJBD/WT-HA	5.4±3.4 n=3
delD/WT-HA	1.1±0.3 n=3

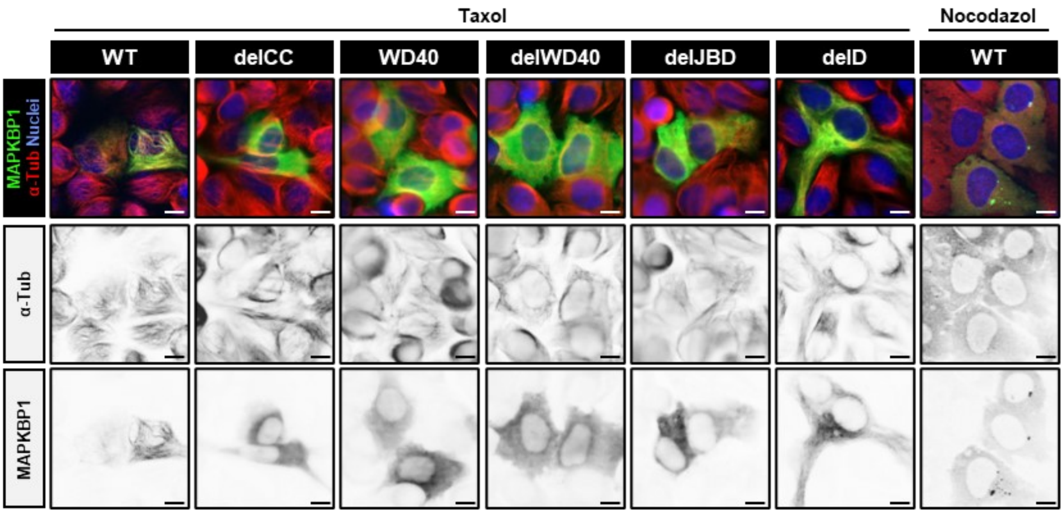

Figure S7: Immunofluorescence microscopy of HeLa cells transiently transfected with GFP-MAPKBP1 wild-type or deletion variants and treated with Taxol or Nocodazol, respectively. Microtubules were stained with anti-α-tubulin primary antibody (GFP-MAPKBP1: green, α-tubulin: red, nuclei: blue). Scale bars equal 10 µm.

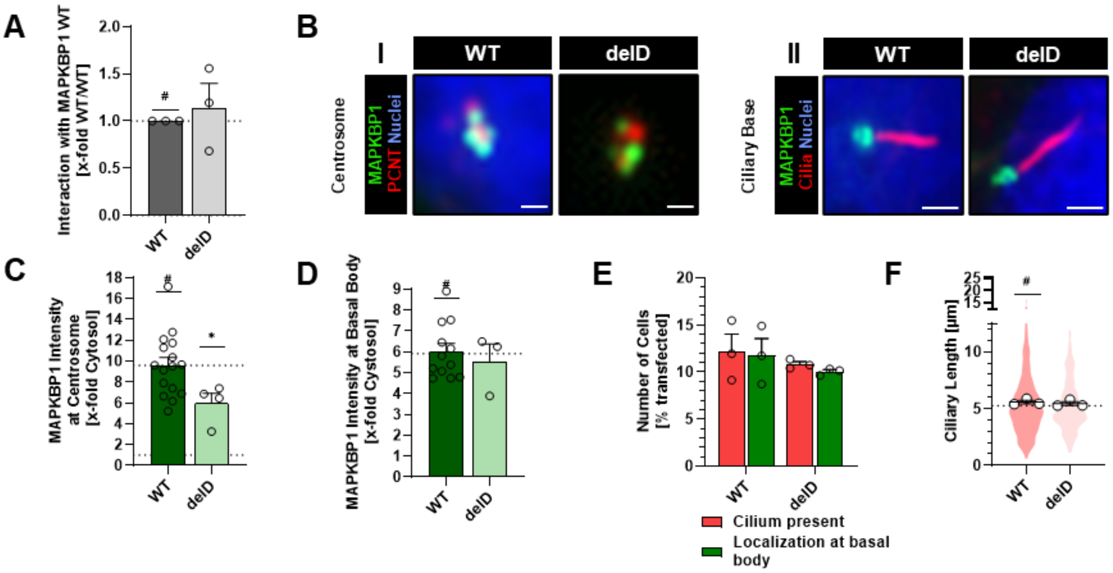

Figure S8: Characterization of MAPKBP1-delD variant**. (A)** Co-immunoprecipitation (Co-IP) of HA-tagged MAPKBP1 wild-type and DDK-tagged delD variant overexpressed in HEK293T cells. Cell lysates and Co-IP eluates were analyzed with western blots using anti-HA and anti-DDK antibodies. Analysis of homodimerization efficiency by quantification of western blot bands. **(B)** Immunofluorescence microscopy of HeLa (I) and H69 (II) cells transiently transfected with GFP-MAPKBP1 wild-type or delD

variants showing centrosomal (I) and ciliary basal body (II) localization. Centrosomes and primary cilia were stained with anti-pericentrin (PCNT) or acetylated α-tubulin antibodies, respectively (GFP-MAPKBP1: green, PCNT/acetylated α-tubulin : red, nuclei: blue). Scale bars equal 1 µm. **(C)** Quantification of GFP intensity at the centrosome in HeLa cells transiently transfected with GFP-MAPKBP1-WT or delD variant, respectively. **(D)** Quantification of GFP intensity at the basal body of primary cilia in H69 cells transiently transfected with GFP-MAPKBP1-WT or delD variant, respectively. **(E)** Quantification of transfected H69 cells presenting a primary cilium (red) and cells with GFP-MAPKBP1 localized at the basal body (green). **(F)** Ciliary length of H69 cells transiently transfected with GFP-MAPKBP1-WT or delD variant. Black bordered white dots represent the mean of each independent experiment. Violin plots represent the sum of all individual datapoints. All data represent the mean (± SEM) of at least three independent experiments. #: reference for statistical testing.

Table S8: Co-immunoprecipitation (Co-IP) of GFP-tagged JNK2 and DDK-tagged MAPKBP1 variants overexpressed in HEK293T cells. Analysis of interaction efficiency by quantification of western blot bands. Data are mean±SEM; JNK2/WT homodimerization was set to 100% in each individual experiment.

JNK2 interaction	interaction with JNK2 [% JNK2/WT]
JNK2/WT-DDK	100.0±0.0 n=5
JNK2/delD-DDK	58.6±20.6 n=4
JNK2/delJBD-DDK	34.0±10.5 n=5
JNK2/delCC-DDK	68.7±13.8 n=3
JNK2/untransfected	2.9±2.9 n=3

Table S9: Quantification of JNK2-intensity in the nucleus in relation to the cytosol depending on the co-transfected MAPKBP1 variant. n=number of independent experiments. Data are mean±SEM and were normalized to JNK2 single transfected (set to 100%) and JNK2+WT (set to 0%) co-transfected cells.

JNK2 interaction	intensity nucleus/cytosol [%]
JNK2	100.0±0.0 n=4
JNK2+WT	0.0±0.0 n=4
JNK2+delD	79.0±3.1 n=3
JNK2+delJBD	116.3±7.1 n=3
JNK2+delCC	10.1±13.6 n=3
JNK2+WD40	76.0±3.5n=3
JNK2+delWD40	-9.1±10.3 n=3

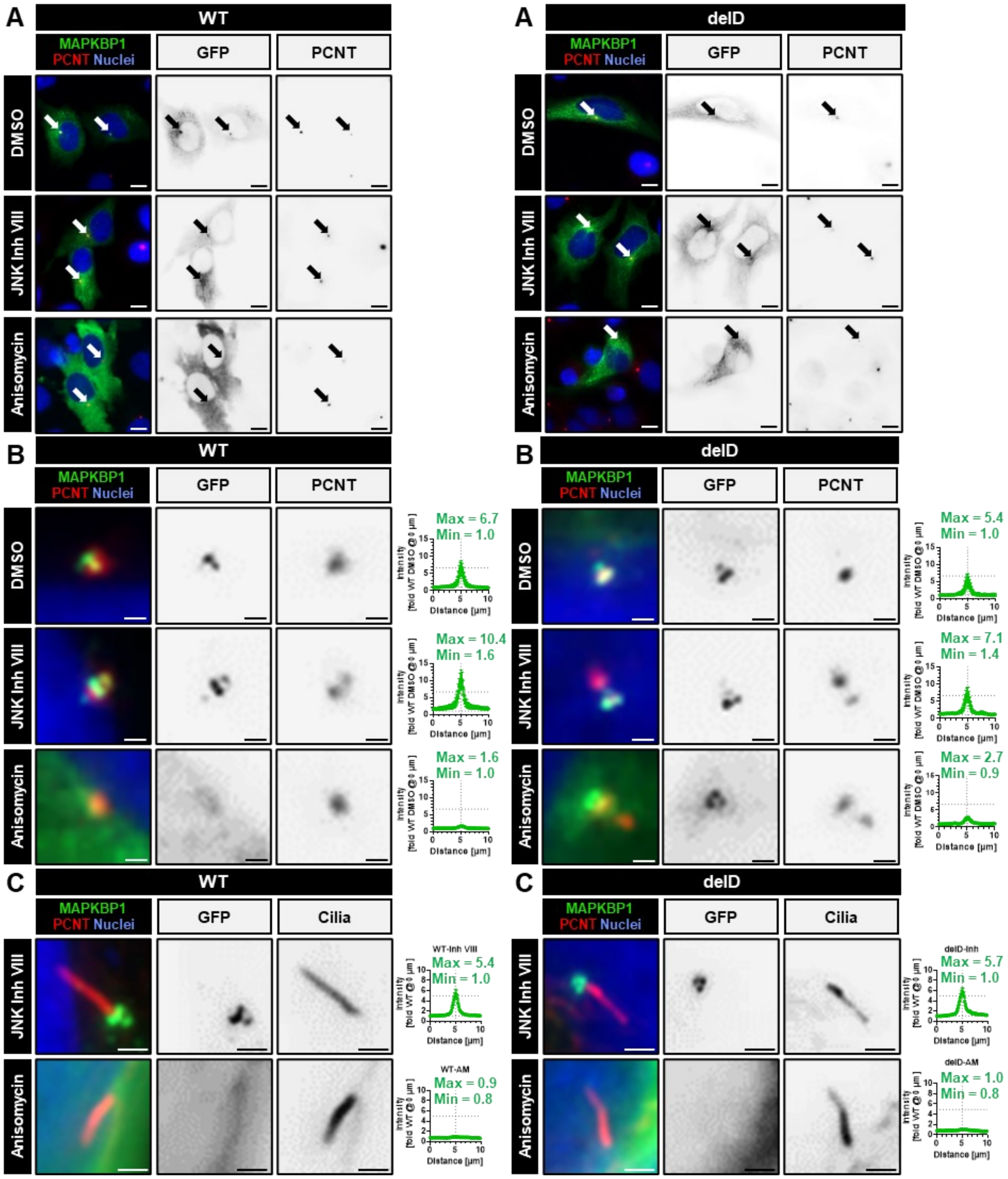

Figure S9: Immunofluorescence microscopy showing overall intracellular **(A),** centrosomal **(B)** and ciliary **(C)** localization of GFP-tagged MAPKBP1 wild-type in transiently transfected HeLa (A,B) or H69 (C) cells upon either inhibition (JNK Inh VIII) or activation (anisomycin, AM) of JNK signaling pathway. **(A,B)** GFP-MAPKBP1: green, PCNT: red, nuclei: blue. **(C)** (GFP-MAPKBP1: green, acetylated α-tubulin: red, nuclei: blue. Scale bars equal 10 µm (A); 1 µm (B); 2 µm (C). **(B)** Quantification of GFP intensity at the centrosome in HeLa cells transiently transfected with GFP-MAPKBP1 wild-type or delD variant treated with either JNK Inh VIII or anisomycin (AM), respectively. Data are mean ± SEM of three independent experiments normalized to GFP-MAPKBP1-WT treated with DMSO (set to 1). **(C)** Quantification of GFP intensity at the primary cilium basal body in H69 cells transiently transfected with GFP-MAPKBP1 wild-type or delD variant treated with either JNK Inh VIII or anisomycin (AM), respectively. Data are mean ± SEM of three independent experiments normalized to GFP-MAPKBP1-WT treated with DMSO (set to 1).

Table S10: GFP intensities at the centrosome of HeLa cells and the basal body of H69 cells transiently transfected with GFP-MAPKBP-WT or delD upon either inhibition (JNK Inh VIII) or activation (anisomycin = AM) of JNK signaling pathway. Data are mean±SEM. n=number of independent experiments (minimum cell number measured in each experiment)

variant + treatment	intensity at centrosome [x-fold cytosol; cytosol WT + DMSO set to 1]	intensity at basal body [x-fold cytosol; cytosol WT + DMSO set to 1]	cilium present [% transfected cells]	localization at basal body [% transfected cells]]
WT + DMSO	7.1±0.6 n=5 (18)	4.9±0.5 n=3 (19)	9.0±1.2 n=3 (99)	8.2±0.8 n=3 (7)
WT + Jnk Inh VIII	6.2±0.5 n=4 (8)	5.4±0.7 n=3 (21)	15.6±2.5 n=3 (98)	14.3±2.6 n=3 (14)
WT + AM	1.9±0.4 n=3 (41)	1.3±0.1 n=3 (21)	7.1±1.0 n=3 (117)	0.2±0.2 n=3 (6)
delD + Jnk Inh VIII	4.9±0.5 n=3 (18)	5.7±0.7 n=3 (20)	12.7±1.3 n=3 (113)	11.2±1.6 n=3 (15)
delD + AM	3.2±0.8 n=3 (25)	1.3±0.1 n=3 (20)	10.7±2.2 n=3 (115)	0.6±0.3 n=3 (11)

Table S11: Quantification of MAPKBP1 wild-type in cell lysates of HEK293T cells treated with either DMSO, JNK Inh VIII or anisomycin (AM), respectively (normalized to the amount in cells treated with DMSO, set to 100%). Co-immunoprecipitation to quantify homodimerization of MAPKBP1 wild-type depending on treatment with either DMSO, JNK Inh VIII or anisomycin (AM), respectively (normalized to homodimerization with DMSO, set to 100%). Data are mean±SEM.

treatment	amount in cell lysate [%]	homodimerization [%]
DMSO	100.0±0.0 n=4	100.0±0.0 n=4
Jnk Inh VIII	67.2±16.0 n=4	102.7±0.0 n=4
AM	176.2±45.0 n=4	83.7±30.1 n=4

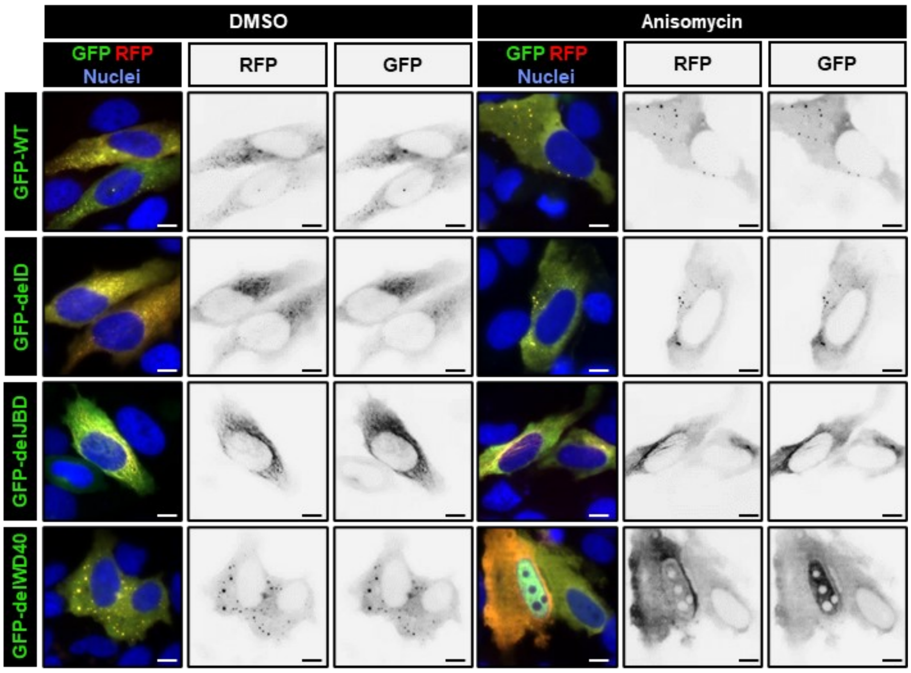

Figure S10: Immunofluorescence microscopy of HeLa cells transiently co-transfected with RFP-tagged MAPKBP1 wild-type and GFP-tagged MAPKBP1 deletion variants. Images show influence of JNK activation (anisomycin) on homodimerization and cellular localization (RFP-MAPKBP1 wild-type: red; GFP-MAPKBP1 variant: green, nuclei: blue). Scale bars equal 10 µm.

Table S12: Immunofluorescence microscopy of HeLa cells transiently co-transfected with RFP-MAPKBP1 wild-type and GFP-MAPKBP1 variants. Quantification of cellular localization pattern depending on JNK signaling activation (anisomycin = AM). Data are mean±SEM [% transfected cells]

		DMSO				AM			
		**GFP-WT**	**GFP-delD**	**GFP-delJBD**	**GFP-delWD40**	**GFP-WT**	**GFP-delD**	**GFP-delJBD**	**GFP-delWD40**
**RFP-WT**	centrosomal	41.1±13.1 n=3	29.0±7.8 n=3	31.2±7.6n=3	34.0±18.9 n=3	7.8±0.4 n=3	9.3±4.7 n=3	19.3±2.6 n=3	0.8±0.8 n=3
	punctate	53.3±7.1 n=3	59.3±14.4 n=3	2.0±2.0 n=3	74.8±12.2n=3	67.3±22.6n=3	60.2±9.2 n=3	7.2±4.0 n=3	8.2±2.9 n=3
	filamentous	51.5±7.4 n=3	61.2±5.6 n=3	84.5±8.2 n=3	6.2±3.3 n=3	3.7±2.1 n=3	13.0±1.0 n=3	52.9±11.1 n=3	0.0±0.0 n=3
	cytosolic	5.3±2.9 n=3	18.8±9.5 n=3	23.3±13.2 n=3	22.0±5.1 n=3	58.1±16.3 n=3	69.1±7.4 n=3	76.7±7.2 n=3	98.9±1.1 n=3
	nuclear	0.0±0.0 n=3	0.0±0.0 n=3	0.0±0.0 n=3	1.0±1.0 n=3	0.0±0.0 n=3	0.0±0.0 n=3	0.0±0.0 n=3	1.1±1.1 n=3
**GFP-variant**	centrosomal	41.1±13.1 n=3	29.0±7.8 n=3	31.2±7.6 n=3	23.6±8.6 n=3	7.8±0.4 n=3	9.3±4.7 n=3	14.3±3.2 n=3	0.8±0.8 n=3
	punctate	53.3±7.1 n=3	59.3±14.4 n=3	2.0±2.0 n=3	74.8±12.2 n=3	67.3±22.6 n=3	60.2±9.2 n=3	10.8±5.5 n=3	8.2±2.9 n=3
	filamentous	51.5±7.4 n=3	61.2±5.6 n=3	68.5±9.4 n=3	6.2±3.3 n=3	3.7±2.1 n=3	13.0±1.0 n=3	35.5±9.9 n=3	0.0±0.0 n=3
	cytosolic	5.3±2.9 n=3	18.8±9.6 n=3	98.9±1.1 n=3	96.7±3.3 n=3	66.8±11.2 n=3	69.1±7.4 n=3	99.5±0.5 n=3	100.0±0.0 n=3
	nuclear	0.0±0.0 n=3	0.0±0.0 n=3	0.0±0.0 n=3	11.0±6.6 n=3	0.0±0.0 n=3	0.0±0.0 n=3	1.1±1.1n=3	11.8±4.8 n=3

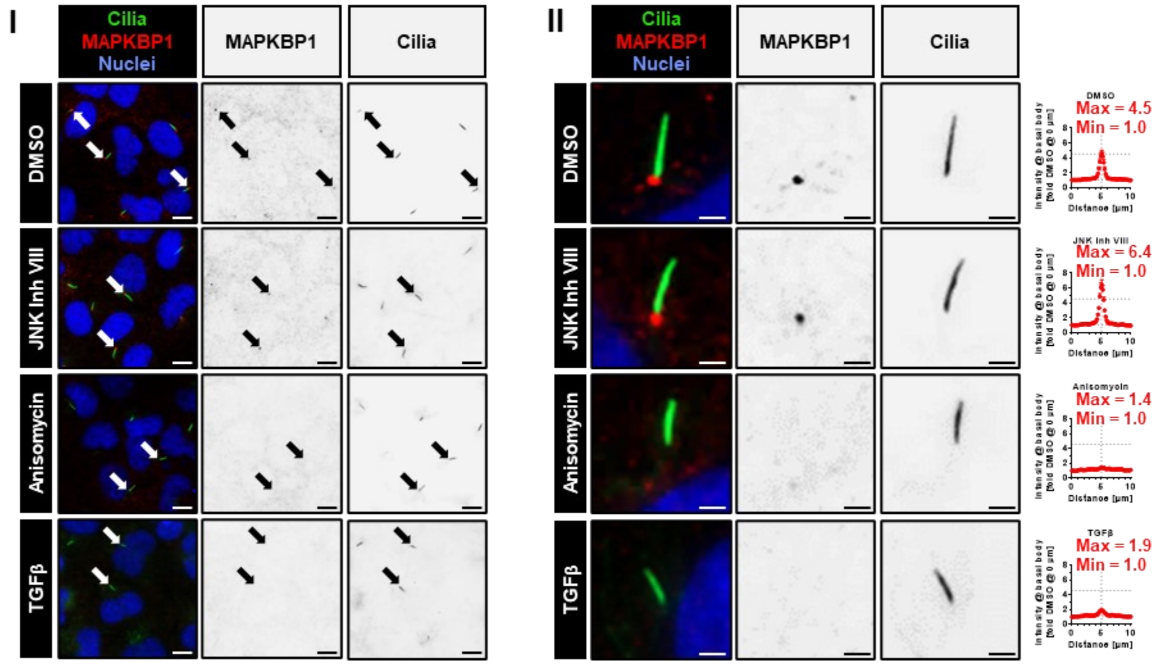

Figure S11: Immunofluorescence microscopy of RPE1 cells treated with either DMSO, JNK Inh VIII, anisomycin (AM) or TGFβ. Endogenous MAPKBP1 was stained with anti-MAPKBP1 antibody (MAPKBP1: red, acetylated α-tubulin: green, nuclei: blue). Scale bars equal 10 µm (I); 2 µm (II). Quantification of MAPKBP1 intensity at the basal body. Data are mean ± SEM of at least three independent experiments normalized to DMSO (set to 1).

Table S13: MAPKBP1 intensities at basal body and ciliary length of untransfected RPE1 cells upon either inhibition (JNK Inh VIII) or activation (anisomycin=AM/TGFβ) of JNK signaling pathway. Data are mean±SEM. n=number of independent experiments (minimum cell number measured in each experiment)

treatment	intensity at basal body [x-fold cytosol; DMSO cytosol set to 1]	ciliary length [µm]
DMSO	4.5±0.5 n=6 (48)	4.0±0.1 n=6 (52)
Jnk Inh VIII	6.4±0.7 n=3 (50)	4.0±0.2 n=3 (47)
AM	1.4±0.2 n=3 (44)	3.5±0.1 n=3 (50)
TGFβ	2.0±0.3 n=3 (52)	3.3±0.0 n=3 (100)

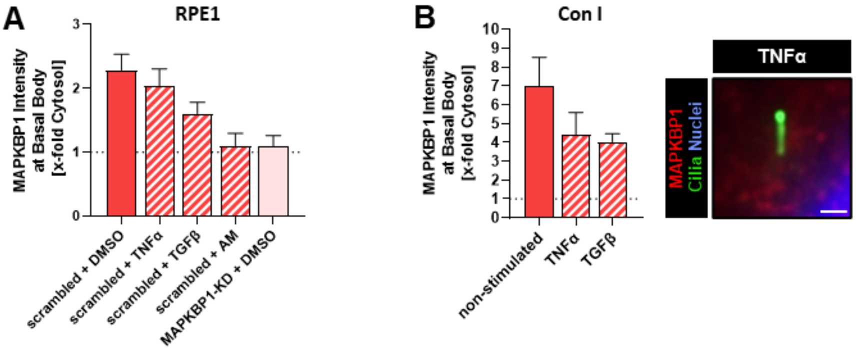

Figure S12: Quantification of MAPKBP1 intensity at the basal body in RPE1 cells **(A)** or control dermal fibroblasts **(B)** upon treatment with TGFβ or TNFα. Endogenous MAPKBP1 was stained with primary anti-MAPKBP1 antibody (MAPKBP1: red, acetylated α-tubulin: green, nuclei: blue). Scale bar equals 2 µm (cilia). Data are mean ± SEM of one example experiment.

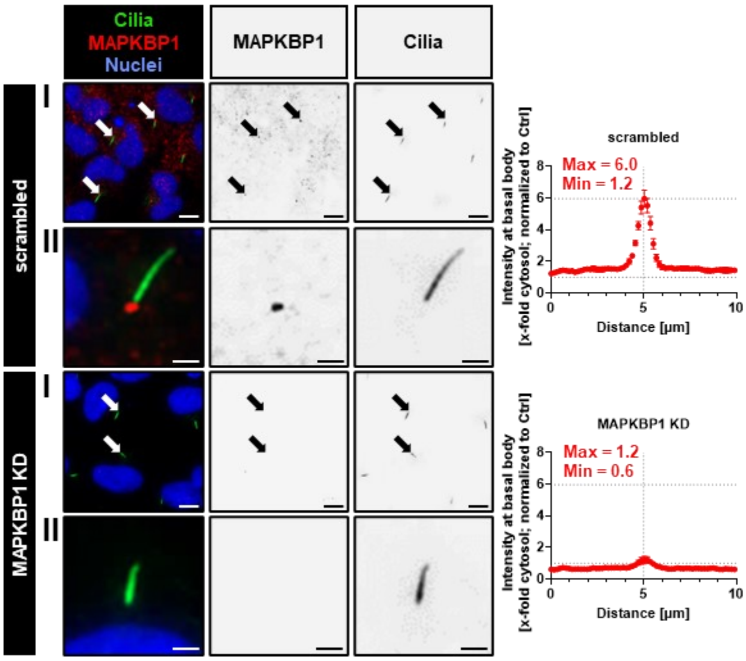

Figure S13: Immunofluorescence microscopy of RPE1 cells transfected with anti-MAPKBP1 siRNA (MAPKBP1-KD) or scrambled siRNA. Endogenous MAPKBP1 was stained with anti-MAPKBP1 antibody (MAPKBP1: red acetylated α-tubulin: green, nuclei: blue). Scale bars equal 10 µm (I); 2 µm (II). (A-B II) Quantification of MAPKBP1 intensity at the basal body. Data are mean ± SEM of three independent experiments normalized to untransfected cells (set to 1).

Table S14: MAPKBP1 intensities and ciliary length of RPE1 cells transfected with either scrambled siRNA or anti-MAPKBP1 control siRNA. Data are mean±SEM. n=number of independent experiments (minimum cell number measured in each experiment)

**variant**	**intensity at basal body [x-fold cytosol; cytosol scrambled set to 1]**	**ciliary length [µm]**
scrambled	4.9±0.6 n=3 (50)	4.2±0.1 n=3 (94)
MAPKBP1-KD	2.0±0.4 n=3 (50)	3.7±0.0 n=3 (98)

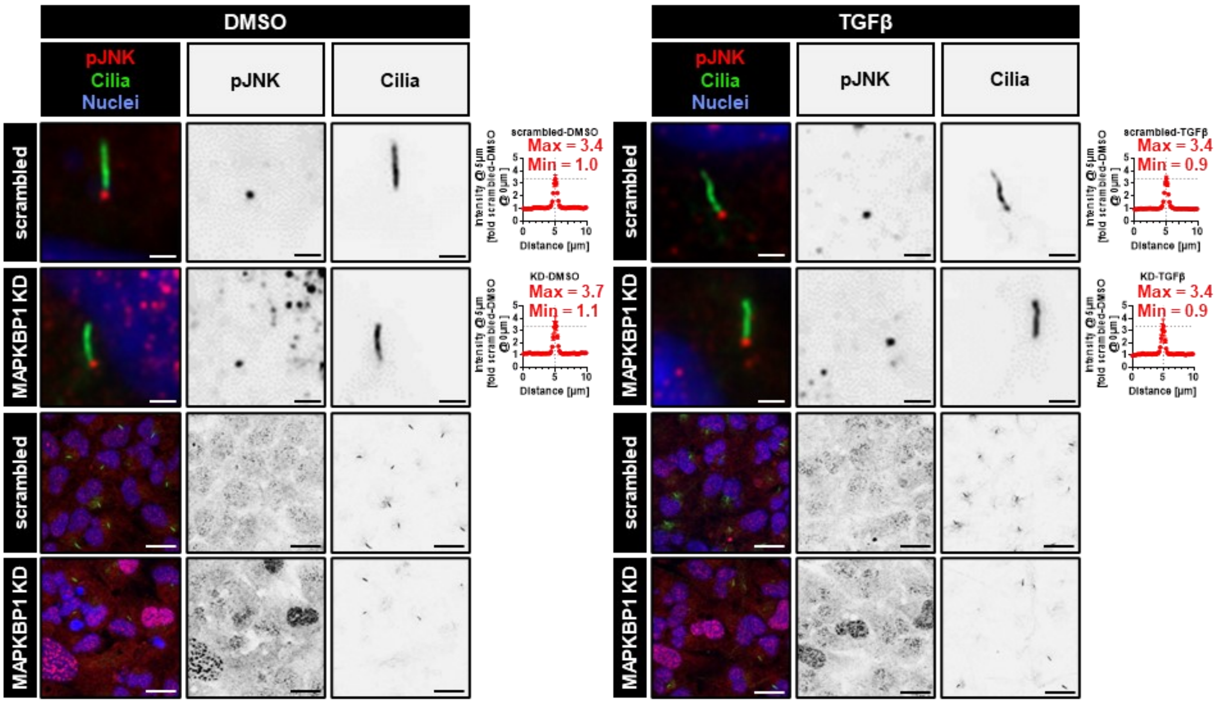

Figure S14: Immunofluorescence microscopy of endogenous pJNK at the basal body of primary cilia and in the nuclei of RPE1 cells transfected with anti-MAPKBP1 siRNA (MAPKBP1-KD) or scrambled siRNA and treated with either DMSO or TGFβ. Endogenous pJNK was stained with anti-pJNK antibody (pJNK: red, acetylated α-tubulin: green, nuclei: blue). Scale bars equal 2 µm (cilia); 10 µm (nuclei). Quantification of pJNK intensity at the basal body depending on TGFβ treatment. Data are mean ± SEM of three independent experiments normalized to cells transfected with scrambled siRNA and treated with DMSO (set to 1).

Table S15: pJNK intensities at basal body and in the nucleus of RPE1 cells transfected with either scrambled siRNA or anti-MAPKBP1 control siRNA treated with either DMSO or TGFβ, respectively. Data are mean±SEM. n=number of independent experiments (minimum cell number measured in each experiment)

**variant + treatment**	**intensity at basal body [x-fold cytosol; cytosol scrambled set to 1]**	**nuclear intensity [x-fold scrambled]**
scrambled + DMSO	3.4±0.3 n=3 (52)	1.0±0.0 n=3 (45)
MAPKBP1-KD + DMSO	3.3±0.2 n=3 (52)	1.4±0.1 n=3 (53)
scrambled + TGFβ	3.6±0.3 n=3 (51)	1.0±0.0 n=3 (53)
MAPKBP1-KD + TGFβ	3.6±0.4 n=3 (51)	1.3±0.1 n=3 (55)

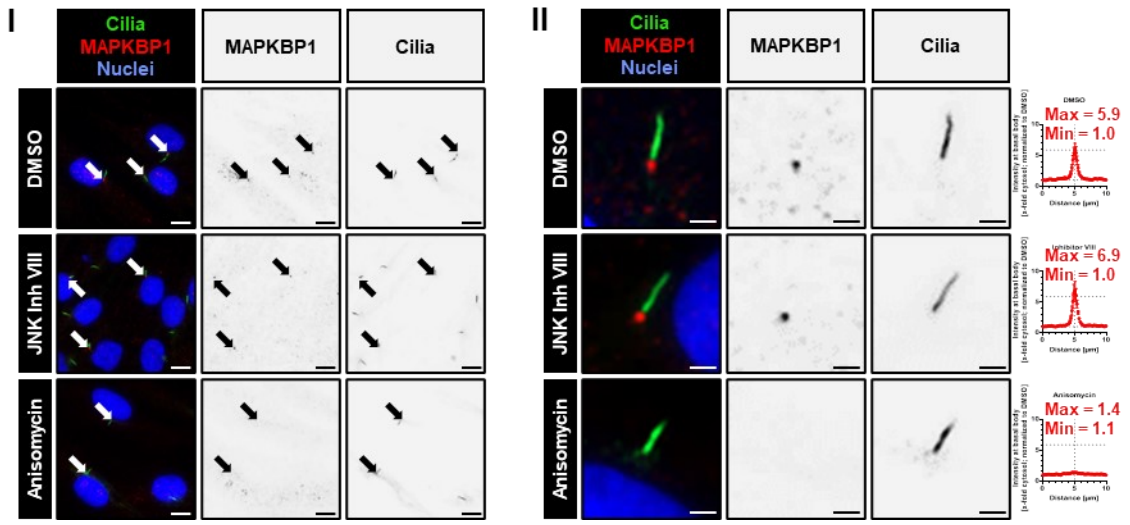

Figure S15: Immunofluorescence microscopy of primary fibroblasts of a healthy control individual treated with either DMSO, JNK Inh VIII, anisomycin (AM). Endogenous MAPKBP1 stained with anti-MAPKBP1 antibody (MAPKBP1: red, acetylated α-tubulin: green, nuclei: blue). Scale bars equal 10 µm (I); 2 µm (II). Quantification of MAPKBP1 intensity at the basal body. Data are mean ± SEM of three independent experiments normalized to cells treated with DMSO (set to 1).

Table S16: MAPKBP1 intensities at basal body and ciliary length of healthy control dermal fibroblasts upon either inhibition (JNK Inh VIII) or activation (anisomycin = AM) of JNK signaling pathway. Data are mean±SEM. n=number of independent experiments (minimum cell number measured in each experiment)

treatment	intensity at basal body [x-fold cytosol; cytosol DMSO set to1]	ciliary length [µm]
DMSO	5.9±1.1 n=3 (39)	3.9±0.3 n=3 (46)
Jnk Inh VIII	6.7±0.2 n=3 (35)	3.5±0.2 n=4 (28)
AM	1.4±0.1 n=3 (30)	3.2±0.1 n=4 (49)

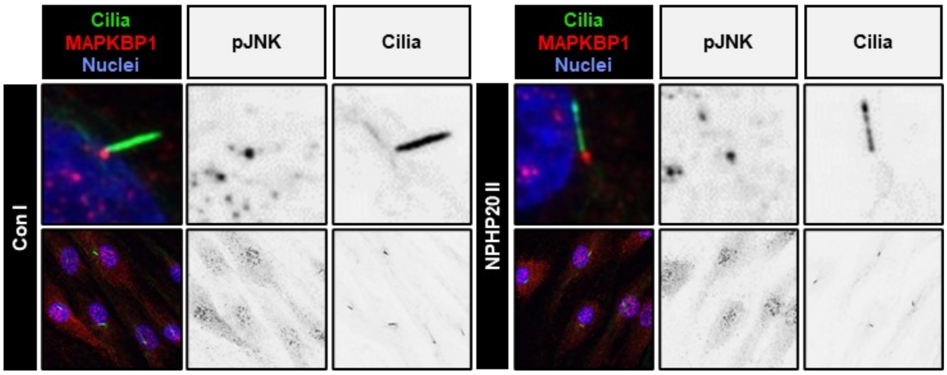

Figure S16: Immunofluorescence microscopy of endogenous pJNK at the basal body of primary cilia and in the nuclei in primary fibroblasts from a healthy control individual and a NPHP20 patient. Endogenous pJNK was stained with primary anti-pJNK antibody (pJNK: red, acetylated α-tubulin: green, nuclei: blue). Scale bars equal 2 µm (cilia); 10 µm (nuclei).

Table S17: pJNK intensities at the basal body and in the nucleus in dermal fibroblasts of healthy control individuals and NPHP20 patients. Each variant was analyzed in three independent experiments with at least 7 cells measured. Data are mean±SEM and were normalized to Con I (set to 100% or 1).

cells	intensity at basal body [% cytosol]	intensity in nucleus [x-fold Con I]
controls	101.5±4.4 n=5	1.0±0.4 n=7
patients	76.5±3.5 n=7	1.1±0.1 n=9

Table S18: Ciliary length of primary cells derived from healthy control individuals I and II and NPHP20 patients I, II and V with and without treatment with 250 nM Cytochalasin D. Data are mean±SEM. n=number of independent experiments (minimum cell number measured in each experiment = 96).

cells	Ciliary length w/o Cyto D [µm]	Ciliary length + Cyto D [µm]
controls	4.7±0.1 n≥3	4.5±0.1 n=3
patients	4.0±0.1 n≥3	4.4±0.1 n=3

STROBE Statement—checklist of items that should be included in reports of observational studies

	Item No.	Recommendation	Page No.	Relevant text from manuscript
**Title and abstract**	1	(*a*) Indicate the study's design with a commonly used term in the title or the abstract	Does not apply	
		(*b*) Provide in the abstract an informative and balanced summary of what was done and what was found	p.2	systematic investigation of all published and newly identified cases
**Introduction**				
Background/rationale	2	Explain the scientific background and rationale for the investigation being reported	p.3	phenotypic differences; almost unknown physiological function
Objectives	3	State specific objectives, including any prespecified hypotheses	p.3	investigating ciliary consequences of MAPKBP1 patient variants
**Methods**				
Study design	4	Present key elements of study design early in the paper	p.6	Combined clinical endpoint analysis
Setting	5	Describe the setting, locations, and relevant dates, including periods of recruitment, exposure, follow-up, and data collection	p.6	16 patients from 12 families with biallelic loss-of-function variants in MAPKBP1 have been reported; comparative data from one of the largest NPH registries
Participants	6	(*a*) *Cohort study*—Give the eligibility criteria, and the sources and methods of selection of participants. Describe methods of follow-up<br>*Case-control study*—Give the eligibility criteria, and the sources and methods of case ascertainment and control selection. Give the rationale for the choice of cases and controls<br>*Cross-sectional study*—Give the eligibility criteria, and the sources and methods of selection of participants	p.6	patients with MAPKBP1-associated kidney disease; comparative data from one of the largest NPH registries
		(*b*) *Cohort study*—For matched studies, give matching criteria and number of exposed and unexposed		

		Case-control study—For matched studies, give matching criteria and the number of controls per case		
Variables	7	Clearly define all outcomes, exposures, predictors, potential confounders, and effect modifiers. Give diagnostic criteria, if applicable	p.6;	median age kidney failure; mean age kidney failure; ; extrarenal manifestations
Data sources/ measurement	8*	For each variable of interest, give sources of data and details of methods of assessment (measurement). Describe comparability of assessment methods if there is more than one group	Does not apply	
Bias	9	Describe any efforts to address potential sources of bias	Does not apply	
Study size	10	Explain how the study size was arrived at	See point 6	

Continued on next page

Quantitative variables	11	Explain how quantitative variables were handled in the analyses. If applicable, describe which groupings were chosen and why	Does not apply	
Statistical methods	12	(*a*) Describe all statistical methods, including those used to control for confounding	See point 6	
		(*b*) Describe any methods used to examine subgroups and interactions	Does not apply	
		(*c*) Explain how missing data were addressed	Does not apply	
		(*d*) *Cohort study*—If applicable, explain how loss to follow-up was addressed<br>*Case-control study*—If applicable, explain how matching of cases and controls was addressed<br>*Cross-sectional study*—If applicable, describe analytical methods taking account of sampling strategy	Does not apply	
		(*e*) Describe any sensitivity analyses	Does not apply	
**Results**				
Participants	13*	(a) Report numbers of individuals at each stage of study—eg numbers potentially eligible, examined for eligibility, confirmed eligible, included in the study, completing follow-up, and analysed	p.6; p. S3-5	
		(b) Give reasons for non-participation at each stage	Does not apply	
		(c) Consider use of a flow diagram	Does not apply	
Descriptive data	14*	(a) Give characteristics of study participants (eg demographic, clinical, social) and information on exposures and potential confounders	p.6; p. S3-5	Known pathogenic MAPKBP1 variants found in patients with NPH; Newly identified NPHP20 patient; Genes associated with certain ciliary modules were combined into distinct subgroups
		(b) Indicate number of participants with missing data for each variable of interest	Does not apply	
		(c) *Cohort study*—Summarise follow-up time (eg, average and total amount)	Does not apply	
Outcome data	15*	*Cohort study*—Report numbers of outcome events or summary measures over time	p.6; p. S5	Phenotypic comparison of NPHP20

				patients with aggregate data from the french NPH cohort
		Case-control study—Report numbers in each exposure category, or summary measures of exposure	Does not apply	
		Cross-sectional study—Report numbers of outcome events or summary measures	Does not apply	
Main results	16	(*a*) Give unadjusted estimates and, if applicable, confounder-adjusted estimates and their precision (eg, 95% confidence interval). Make clear which confounders were adjusted for and why they were included	p.6, p.S5	
		(*b*) Report category boundaries when continuous variables were categorized	Does not apply	
		(*c*) If relevant, consider translating estimates of relative risk into absolute risk for a meaningful time period	Does not apply	

Continued on next page

Other analyses	17	Report other analyses done—eg analyses of subgroups and interactions, and sensitivity analyses	See point 15	
**Discussion**				
Key results	18	Summarise key results with reference to study objectives	p.10	Clinical endpoint analysis of kidney failure revealed a later onset of MAPKBP1-associated kidney disease compared to all other forms of NPH
Limitations	19	Discuss limitations of the study, taking into account sources of potential bias or imprecision. Discuss both direction and magnitude of any potential bias	Does not apply	
Interpretation	20	Give a cautious overall interpretation of results considering objectives, limitations, multiplicity of analyses, results from similar studies, and other relevant evidence	p.10	
Generalisability	21	Discuss the generalisability (external validity) of the study results	Does not apply	
**Other information**				
Funding	22	Give the source of funding and the role of the funders for the present study and, if applicable, for the original study on which the present article is based	p.13	

*Give information separately for cases and controls in case-control studies and, if applicable, for exposed and unexposed groups in cohort and cross-sectional studies.

Note: An Explanation and Elaboration article discusses each checklist item and gives methodological background and published examples of transparent reporting. The STROBE checklist is best used in conjunction with this article (freely available on the Web sites of PLoS Medicine at http://www.plosmedicine.org/, Annals of Internal Medicine at http://www.annals.org/, and Epidemiology at http://www.epidem.com/). Information on the STROBE Initiative is available at www.strobe-statement.org.

Supplemental references

[S1] Zehorai E, Seger R. Beta-like importins mediate the nuclear translocation of mitogen-activated protein kinases. Mol Cell Biol 2014;34(2):259–70. https://doi.org/10.1128/MCB.00799-13.
